# Autism spectrum disorder related phenotypes in a mouse model lacking the neuronal actin binding protein profilin 2

**DOI:** 10.3389/fncel.2025.1540989

**Published:** 2025-02-26

**Authors:** Walter Witke, Marina Di Domenico, Laura Maggi, Alessia Di Nardo, Valentin Stein, Pietro Pilo Boyl

**Affiliations:** ^1^Institute of Genetics, University of Bonn, Bonn, Germany; ^2^Dipartimento di Fisiologia e Farmacologia, Research Center of Neuroscience “CRiN-Daniel Bovet”, University Sapienza Rome, Rome, Italy; ^3^Biogen Inc., Cambridge, MA, United States; ^4^Institute of Physiology II, Faculty of Medicine, University of Bonn, Bonn, Germany

**Keywords:** profilin 2, glutamatergic neuron hyperactivity, excitation/inhibition balance, autism spectrum disorder, seizures

## Abstract

**Introduction:**

Profilin 2 (PFN2) is an actin binding protein highly expressed in the brain that participates in actin dynamics. It has been shown in vitro and in vivo that in neurons it functions both post-synaptically to shape and maintain dendritic arborizations and spine density and plasticity, as well as pre-synaptically to regulate vesicle exocytosis. PFN2 was also found in protein complexes with proteins that have been implicated in or are causative of autism spectrum disorder.

**Methods:**

We employ a genetically engineered knock-out mouse line for *Pfn2* that we previously generated to study the mouse social, vocal and motor behavior in comparison to wild type control littermates. We also study neuronal physiology in the knock-out mouse model by means of cellular and field electrophysiological recordings in cerebellar Purkinje cells and in the Schaffer collaterals. Lastly, we study anatomical features of the cerebellum using immunofluorescence stainings.

**Results:**

We show that PFN2 deficiency reproduces a number of autistic-like phenotypes in the mouse, such as social behavior impairment, stereotypic behavior, altered vocal communication, and deficits in motor performance and coordination. Our studies correlate the behavioral phenotypes with increased excitation/inhibition ratio in the brain, due to brain-wide hyperactivity of glutamatergic neurons and increased glutamate release not compensated by enhanced GABAergic neurotransmission. Consequently, lack of PFN2 caused seizures behavior and age-dependent loss of cerebellar Purkinje cells, comorbidities observed in a subset of autistic patients, which can be attributed to the effect of excessive glutamatergic neurotransmission.

**Discussion:**

Our data directly link altered pre-synaptic actin dynamics to autism spectrum disorder in the mouse model and support the hypothesis that synaptic dysfunctions that asymmetrically increase the excitatory drive in neuronal circuits can lead to autistic-like phenotypes. Our findings inspire to consider novel potential pathways for therapeutic approaches in ASD.

## 1 Introduction

Since the last two decades the relevant role of the actin cytoskeleton for the development and function of the nervous system has been extensively studied. Both growth cone dynamics and neurite extension (for a review see Omotade et al., 2017), as well as synaptic plasticity (for a review see Cingolani and Goda, 2008), have been shown to depend on actin dynamics. Actin binding proteins (ABP) are key factors that convey extracellular cues to actin cytoskeleton rearrangements. Only a few ABPs have been genetically linked to neurodevelopmental or neurological disorders in humans, either through copy number variation (CNV) or single nucleotide variation (SNV) and microdeletions, including: inverted formin 2 (IFN2) (Boyer et al., 2011; Mademan et al., 2013), profilin 1 (PFN1) (Wu et al., 2012; Smith et al., 2015), the cytoplasmic FMR1 interacting protein 1 and 2 (CYFIP1 and CYFIP2) (Oguro-Ando et al., 2015; Nakashima et al., 2018; Peng et al., 2018), spectrin beta III (SPTBN2) (Avery et al., 2017), microtubule actin crosslinking factor 1 (MACF1) (Dobyns et al., 2018), dystonin (DST) (Fortugno et al., 2019). The relevance of ABPs in neurodevelopmental and neurophysiological processes has also been assessed in mouse models, which have shown phenotypes ranging from neuronal cell migration impairment to neuronal development defects, as well as to alterations in synaptic maturation, connectivity and plasticity (Soderling et al., 2003; Grove et al., 2004; Bellenchi et al., 2007; Pilo Boyl et al., 2007; Bozdagi et al., 2012; Pathania et al., 2014; Han et al., 2015; Di Domenico et al., 2021; Klemz et al., 2021). Often these mouse models displayed certain anatomical or physiological conditions typical of neurodevelopmental disorders, such as autism spectrum disorder (ASD), intellectual disability, schizophrenia, and epilepsy (Soderling et al., 2003; Bozdagi et al., 2012; Han et al., 2015; Davenport et al., 2019), however, the behavioral phenotypes were mild possibly due to heterozygosity, or to compensatory mechanisms, or to functional redundancy of paralogs, or because deletion of the specific gene occurred too late in brain development.

The *profilin 2* (*Pfn2*) conventional knock-out (ko) mouse model (*Pfn2^−/−^*) is viable and has been already shown to display synaptic, physiological, and behavioral phenotypes typical of neurodevelopmental disorders (Gareus et al., 2006; Pilo Boyl et al., 2007). The *Pfn2* ko mouse model has two distinct features when compared to other ABPs knock-out models: (1) PFN2 function seems to be mainly restricted to regulating actin dynamics at the synapse, both in pre-synaptic boutons and in dendritic spines (Ackermann and Matus, 2003; Pilo Boyl et al., 2007), the neuronal compartment that has been recently proposed to be a hotspot for mental disorders (Yan et al., 2016); and (2) in this mouse model, PFN2 is absent during the entire embryonic development without resulting in pre-natal or juvenile lethality (Pilo Boyl et al., 2007; Di Domenico et al., 2021), as happens for many ABPs knock-out mouse models. The viability of *Pfn2*^−/−^ mice can be explained by the restricted expression and function of PFN2, which only starts in post-mitotic neurons around embryonic day 12.5, and to a partial compensatory effect from its paralog, profilin 1 (Di Domenico et al., 2021). This combination of factors allows the study of the knock-out phenotype, which originates during embryonic development, in adult and aging mice. Functionally PFN2 is a small 14 kDa protein that forms a 1:1 complex with globular (G-) actin (profilactin) and accelerates the ADP to ATP exchange that primes G-actin to polymerization (Gieselmann et al., 1995; Paul and Pollard, 2009). In principle, this allows profilin 2 to coordinate the rate, place, and type of actin polymerization through the interaction with numerous specific actin nucleation and elongation factors (Krause et al., 2003; Faix and Grosse, 2006; Pollard, 2007) via its poly-L-proline (PLP) binding domain.

The human genomic locus 3q25-27, encompassing the *PFN2* gene, has been linked to autism spectrum disorder in two past studies on Finnish families in Scandinavia and the USA (AUTS2, now, due to a nomenclature overlap, renamed AUTS8, OMIM: 607373) (Auranen et al., 2002; Coon et al., 2005), but until now no gene inactivating SNVs, CNVs, or microdeletions affecting specifically the *PFN2* gene have been reported in exome sequencing studies. The absence of a clear association of the ASD phenotype to any of the genes included in the 3q25-27 human locus together with the findings on the specific synaptic function of PFN2 has prompted us to revisit a potential role of profilin 2 in autistic-like behavior. The core ASD traits are defined by the DSM-5 guidelines (American Psychiatric Association, 2013) as: (i) Social interaction deficits; (ii) Repetitive, ritualistic, and stereotyped behavior. In this work we show that the *Pfn2* ko mouse presents with deficits in both these core traits. In addition, we investigate comorbid traits that often accompany the main autistic phenotype, in particular vocal communication, that we studied in the mouse pups, and motor coordination. We find that these behaviors are robustly altered in the *Pfn2^−/−^* mouse model, which could therefore represent an interesting *in vivo* model to study or validate one possible pathway causing autism spectrum disorder.

## 2 Results

### 2.1 Infantile mortality and shorter life expectancy of *Pfn2*^−/−^ mice

**Figure 1:**
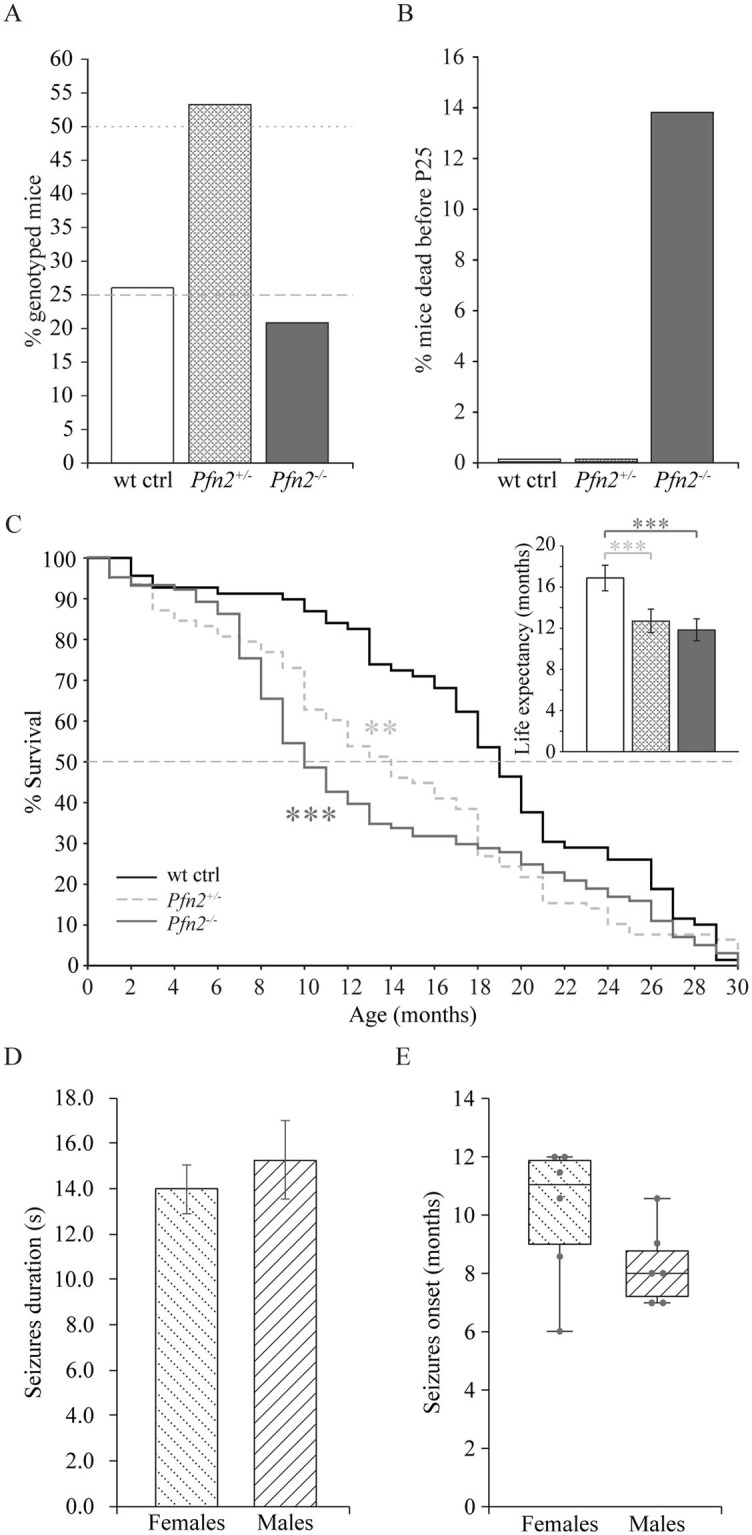
Reduced survival before weaning and shorter life expectancy of *Pfn2*^−/−^ mice. **(A)** In *Pfn2*^+/−^ matings, fewer *Pfn2*^−/−^ offspring mice were found than expected from Mendelian ratio (dashed and dotted gray lines) at weaning (P21-P25). *N* = 47 wt, *N* = 97 *Pfn2*^+/−^, and *N* = 38 *Pfn2*^−/−^ genotyped animals from 24 litters. **(B)** Pre-weaning loss of *Pfn2*^−/−^ pups. Offspring were genotyped at P8, when all genotypes obeyed the Mendelian ratio, and followed until weaning (P25): 14% of *Pfn2*^−/−^ mice died during this period, but no wt and *Pfn2*^+/−^ mice. *N* = 76 wt ctrl, *N* = 178 *Pfn2*^+/−^, *N* = 75 *Pfn2*^−/−^ P8 animals. **(C)** Kaplan–Meier plot of survival data shows higher mortality rate of *Pfn2*^−/−^ mice between 6 and 14 months of age with an intermediate phenotype for *Pfn2*^+/−^ mice (Peto-Prentice generalized Wilcoxon test: *Pfn2*^−/−^ vs. wt ctrl, *p* < 0.001; *Pfn2*^+/−^ vs. wt ctrl, *p* = 0.001). The median survival (dotted line in the graph) was 10 months for *Pfn2*^−/−^ mice, 14 months for *Pfn2*^+/−^ and 19 months for wt controls. Inset shows life expectancy (average life span) for each genotype, 12.9 ± 0.8 months for *Pfn2*^−/−^ mice, 13.8 ± 0.9 months for *Pfn2*^+/−^ and 18.3 ± 1.0 months for wt controls (two-tailed *t-*test: *Pfn2*^−/−^ vs. wt ctrl mice, *p* < 0.0001; *Pfn2*^+/−^ vs. wt ctrl mice, *p* = 0.0008). *N* = 69 wt ctrl, *N* = 78 *Pfn2*^+/−^, *N* = 101 *Pfn2*^−/−^ animals. **(D)** Average duration of seizures in male and female *Pfn2* ko mice. No difference was detected (two-sided *t-*test, *p* = 0.5384). **(E)** Box plot representation of the seizures onset for male and female *Pfn2* ko mice, with single units per group indicated by the gray dots and horizontal bar indicating median. A tendential earlier onset in males was observed (Mann–Whitney *U*-test, *p* = 0.1467). N = 70 *Pfn2^−/−^* mice. **p ≤ 0.01, ***p ≤ 0.001.

*Pfn2* mutants are born in Mendelian ratio but in heterozygous breeding pairs we noted delayed growth of the *Pfn2^−/−^* pups compared to *Pfn^+/−^* and wt control littermates in larger size litters, suggesting a competitive disadvantage during rearing. Genotyping at weaning age (P21-P25) showed a reduced mendelian ratio to about 22% for *Pfn2^−/−^* animals (Figure 1A), pointing to a mortality rate of *Pfn2^−/−^* pups before weaning that was not observed in *Pfn2^+/−^* and wt control littermates (Figure 1B).

These findings prompted us to investigate survival and life expectancy of *Pfn2^−/−^* mice through a standard survival analysis. The Kaplan–Meier plot showed a significant mortality of *Pfn2*^−/−^ mice between 7 and 13 months (Peto-Prentice test, *p* < 0.001), with the median survival halved compared to wt littermate controls (Figure 1C). Interestingly, *Pfn2* heterozygote mice displayed an intermediate phenotype, with significantly reduced survival compared to wt controls (*p* = 0.001), indicating a gene dosage effect on mouse survival. As a consequence, life expectancy (average life span) of both *Pfn2^−/−^* and heterozygote mice was significantly reduced by 30 and 25%, respectively (Figure 1C, inset).

Premature mortality has been reported in autism spectrum disorder (Hirvikoski et al., 2016). One associated cause is epilepsy, in a recent study found in 12% of individuals with autism (Lukmanji et al., 2019). In a small-scale study (N = 70 *Pfn2^−/−^* mice) we observed spontaneous, sensory induced, seizures in 12.5% of *Pfn2^−/−^* mice, irrespective of their sex. The seizures were tonic or tonic–clonic, had an average length of 14 s in females and 15 s in males (Figure 1D), and the mice typically recovered within 60–120 s. The seizures’ onset was tendentially earlier in male mutant mice (8 months) than in females (11 months, Figure 1E). Since the onset of seizures coincides with the period of higher mortality of the *Pfn2^−/−^* mice, we suspect that they might contribute to their reduced life expectancy.

### 2.2 Impaired maternal and social behavior of *Pfn2*^−/−^ mice

The competitive disadvantage of *Pfn2^−/−^* pups during rearing hinted at altered social behavior, either in the relationship of the mutant pups with the mother or with the other littermates for successful breastfeeding. To study sociability more in detail, we addressed two established behaviors in adult mice: maternal behavior of *Pfn2^−/−^* mice toward the pups and social interactions of *Pfn2^−/−^* male mice.

**Figure 2:**
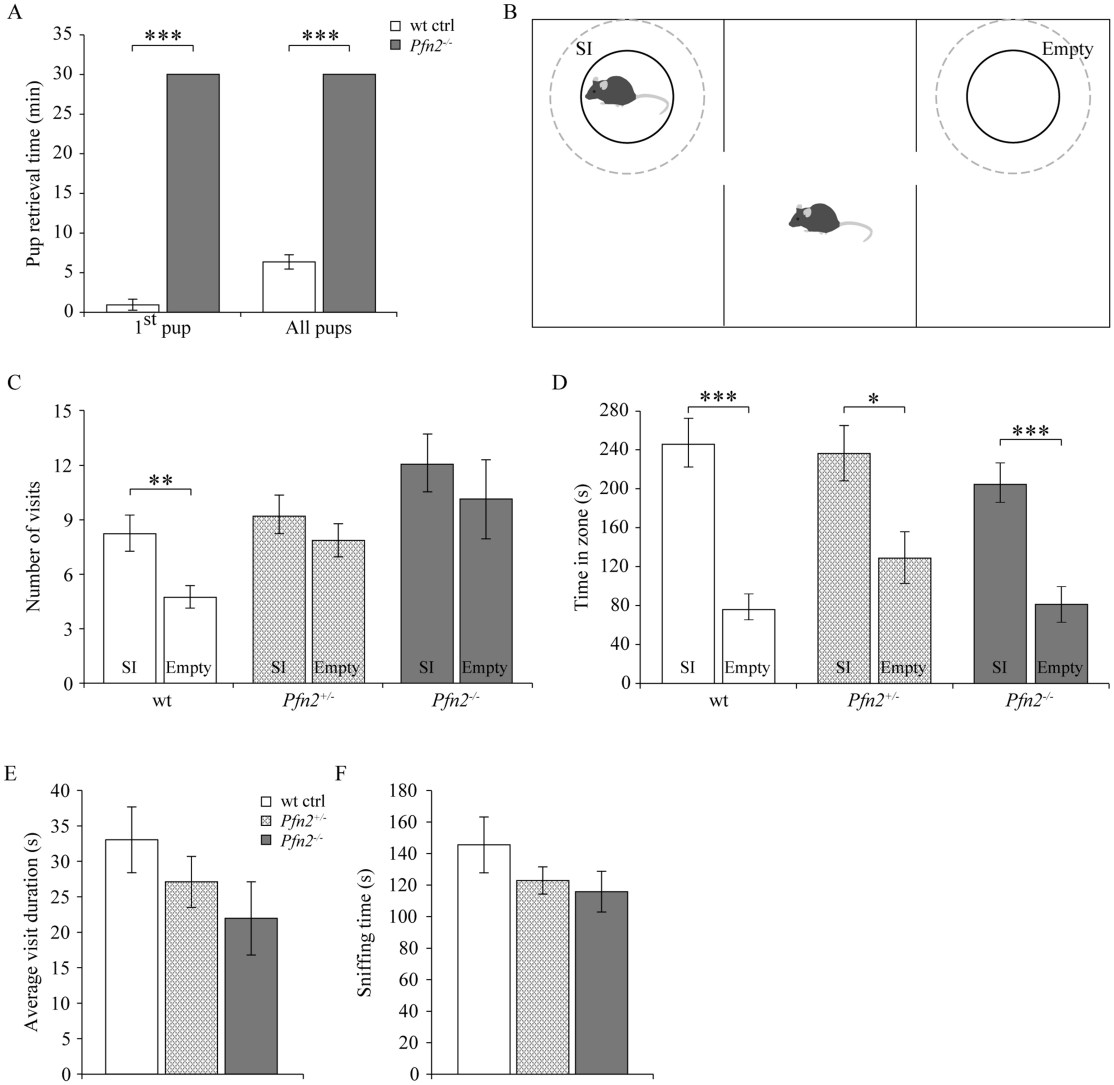
Maternal and social behavior are impaired in *Pfn2*^−/−^ mice. **(A)** Maternal behavior: pup retrieval by *Pfn2*^−/−^ females was completely missing within the experimental time of 30 min, while on average wt control females retrieved the first pup within 1 min (0.8 ± 0.7 min) and the entire litter in 6.2 ± 0.9 min (one-sided *t-*test, *p* < 0.001 for both tests). *N* = 5 wt ctrl and *N* = 7 *Pfn2*^−/−^ females. **(B)** Schematic of the 3-chambered social interaction test. Dashed circles delimit the SI (Social Interaction) zone hosting in our set-up a novel juvenile (~P25) mouse to control for the hyper-excitability of *Pfn2^−/−^* mice, and the Empty zone used for social behavior quantification. **(C)** By number of visits, *Pfn2*^−/−^ and *Pfn2^+/−^* mice showed no significant preference for the SI zone compared to the Empty zone (two-sided *t-*test, *p* = 0.4683 and *p* = 0.3276, respectively) while wt control mice showed a significant preference for the SI zone (two-sided *t-*test *p* = 0.0098), indicating social interaction impairment in mutant and heterozygous mice. **(D)** By the time spent socializing, all genotypes spent more time in the SI zone than in the Empty zone, although *Pfn2*^−/−^ mice showed the least preference. **(E)** On average *Pfn2^−/−^* mice spent less time per visit in the SI zone compared to wt control mice. Heterozygous mice performed in the middle. **(F)** Sniffing in the SI zone was reduced in *Pfn2* mutant mice compared to wt controls. *N* = 8 wt ctrl, *N* = 7 *Pfn2*^+/−^ and *N* = 9 *Pfn2*^−/−^. *p ≤ 0.05, **p ≤ 0.01, ***p ≤ 0.001.

Maternal behavior was found severely affected in *Pfn2^−/−^* mice. Nest building was almost absent in *Pfn2*^−/−^ females (Supplementary Figure 1) and when *Pfn2*^−/−^ mothers were allowed as single parents to rear their litters, all pups were lost irrespectively of the pups’ genotype (Table 1, top part). Interestingly, with the cohabitation of a wt or *Pfn2*^+/−^ father, all litters of *Pfn2*^−/−^ mothers could be rescued and survived. However, only about half of the litters survived when both parents were *Pfn2^−/−^* animals (Table 1, bottom part). One possible reason for the rearing deficit of *Pfn2^−/−^* mothers was a severely compromised pup retrieval behavior: when challenged to retrieve P7 pups dispersed in the cage, wt mothers quickly collected all pups within 7 min back into the nest (Figure 2A). Instead, *Pfn2*^−/−^ mothers never retrieved a single pup within the maximally allowed experimental time (30 min). Thus, *Pfn2*^−/−^ dams completely lacked pup rearing behavior.

**Table 1:** Maternal behavior.

Genotype		Number of litters	Father cohabitation	Litter survival
Mother	Father			
*Pfn2* ^−/−^	wt	7	No	0%
*Pfn2* ^−/−^	*Pfn2* ^+/−^	5	No	0%
*Pfn2* ^−/−^	*Pfn2* ^−/−^	3	No	0%
*Pfn2* ^−/−^	wt	5	Yes	100%
*Pfn2* ^−/−^	*Pfn2* ^+/−^	3	Yes	100%
*Pfn2* ^−/−^	*Pfn2* ^−/−^	7	Yes	45%

Suspecting a general social interaction deficit upon reduction of profilin 2 levels, we applied to adult male mice the Social Interaction paradigm in the 3-chambered maze to assess adult sociability (Figure 2B) (Moy et al., 2007). When social interaction between the adult test mouse and a stranger juvenile was assessed, *Pfn2*^−/−^ mice showed a mild reduction of sociability. Only wt controls showed a significant preference for the Social interaction (SI) zone in terms of number of visits (SI: 8 ± 1 vs. Empty: 5 ± 1), while both *Pfn2*^+/−^ (SI: 9 ± 1 vs. Empty: 8 ± 1) and *Pfn2*^−/−^ (SI: 12 ± 2 vs. Empty: 10 ± 2) mice failed to do so (Figure 2C). Wt control mice also showed the highest preference for the social compartment when measuring the time spent in the SI zone compared to the Empty zone (Figure 2D), although also *Pfn2*^−/−^ and *Pfn2*^+/−^ mice showed some degree of preference for the SI compartment. The average visit duration of *Pfn2*^−/−^ mice to the stranger juvenile mouse was tendentially reduced compared to wt controls (Figure 2E), as well as the average sniffing time (Figure 2F). In addition, the interaction of *Pfn2*^−/−^ mice with the stranger juvenile mouse was qualitatively very different from control mice: direct nose contacts of *Pfn2*^−/−^ mice with the juvenile mouse were rare, with the mutant mice mostly running around and over the metal enclosure. In a previous study (Pilo Boyl et al., 2007) we reported a striking increase of novelty-seeking behavior in *Pfn2*^−/−^ mice, which spent more time than wt control littermates to explore novel objects. We suspect that this phenotype might have masked the social behavior in our set-up, where the juvenile mouse has represented an interesting novel object due to its immature sociability and its motility, triggering a novelty-seeking behavior in the *Pfn2^−/−^* mice.

### 2.3 Increased stereotypic behavior in *Pfn2*^−/−^ mice

**Figure 3:**
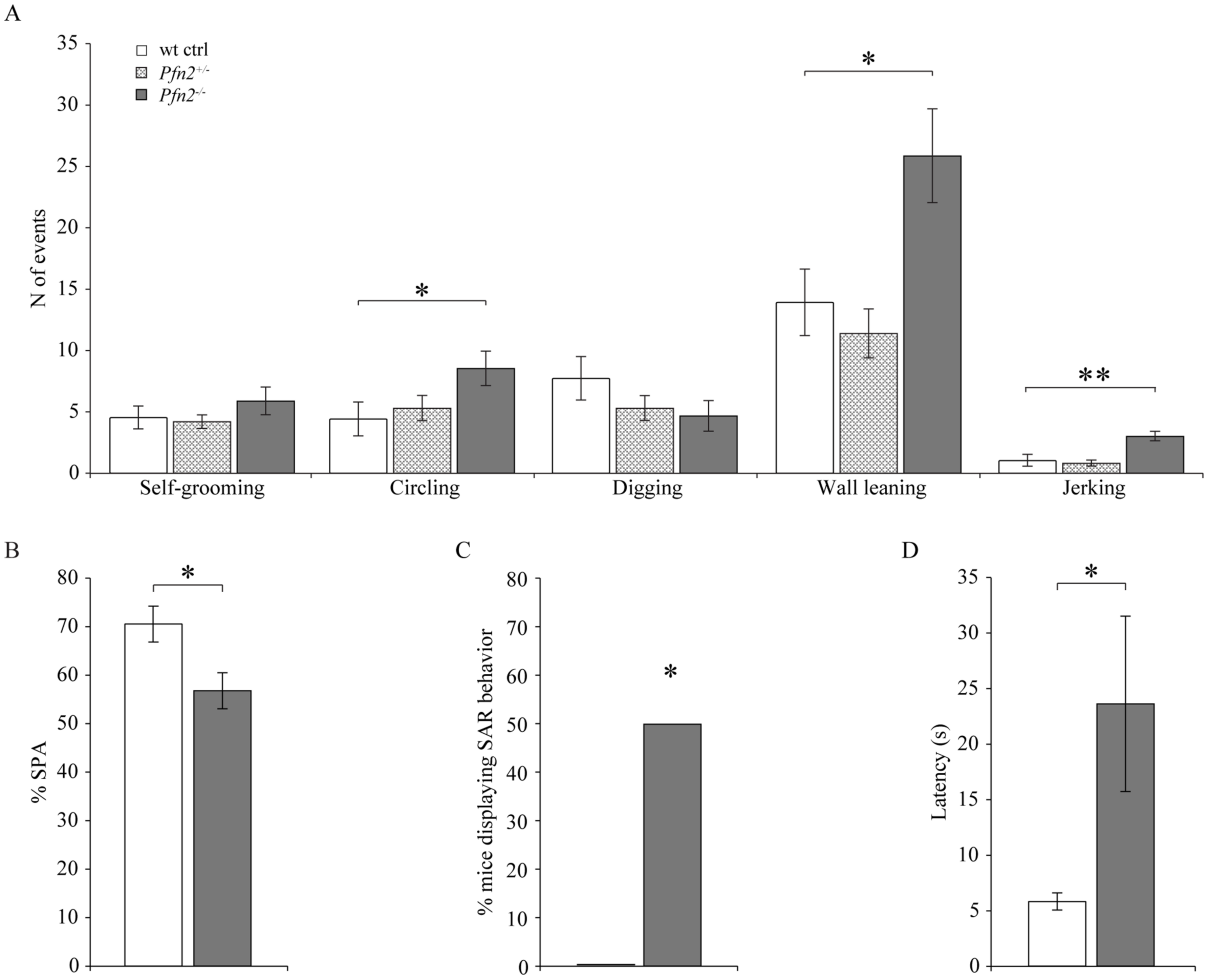
Stereotypic and repetitive behavior is increased in *Pfn2*^−/−^ mice. **(A)** Five stereotypic behaviors were measured after transfer in a novel cage environment. Circling (two-sided *t-*test, *p* = 0.0442), wall leaning (two-sided *t-*test, *p* = 0.0273) and jerking (two-sided *t-*test, *p* = 0.0015) were increased in *Pfn2*^−/−^ mice compared to wt controls, grooming and digging were not changed. *N* = 10 wt ctrl, *N* = 11 *Pfn2*^+/−^ and *N* = 15 *Pfn2*^−/−^ mice. **(B,C)** Y-Maze: spontaneous alternation (SPA) exploration strategy was diminished in *Pfn2*^−/−^ mice compared to wt controls (two-sided *t-*test, *p* = 0.0159) in favor of same arm return (SAR) exploration, performed by 50% of the *Pfn2*^−/−^ mice (one-sample *t-*test, *p* = 0.0122), indicative of a resistance to changes in the mutants. **(D)** Latency to start exploring the maze by entering the first arm was higher in *Pfn2*^−/−^ animals compared to wt controls (two-sided *t-*test, *p* = 0.0409). *N* = 11 wt ctrl, *N* = 10 *Pfn2*^−/−^ animals. *p ≤ 0.05, **p ≤ 0.01.

Ritualistic and repetitive behavior, accompanied by resistance to changes, is the second core symptom that characterizes ASD and can be measured in mouse models for ASD as stereotypic behavior (Peça et al., 2011; Schmeisser et al., 2012). We therefore assessed an array of stereotypic behaviors in control and *Pfn2* mutant mice after transfer into a novel cage. In this context, no significant differences were observed for self-grooming and digging. However, *Pfn2*^−/−^ animals showed significantly higher occurrence of circling, and wall leaning stereotypies compared to *Pfn2*^+/−^ and wt control mice (Figure 3A), indicating a stronger susceptibility to repetitive behavior. Also jerking, a tic-like stereotypic behavior that can have both physiologic or pathologic origin, was significantly increased in *Pfn2*^−/−^ mice (Figure 3A).

Complementary to this general compulsive behavior, we evaluated the resistance to changes in *Pfn2*^−/−^ mice, using a more sophisticated paradigm. For this purpose, we tested the animals in a Y-maze set-up, where mice typically explore the arms of the maze in sequential order (spontaneous alternation, SPA). *Pfn2*^−/−^ mice displayed decreased SPA (Figure 3B), and while in our experiment no control mouse ever returned to the same arm (SAR), about 50% of the tested *Pfn2*^−/−^ mice showed this repetitive behavior (Figure 3C). *Pfn2*^−/−^ mutants also showed a marked deficit in decision making, as suggested by the longer latency to initiate exploration (Figure 3D). These data suggest a propensity to repetitive behavior and an increased resistance to changes in *Pfn2^−/−^* mice.

### 2.4 Altered vocalization pattern in *Pfn2*^−/−^ pups

**Figure 4:**
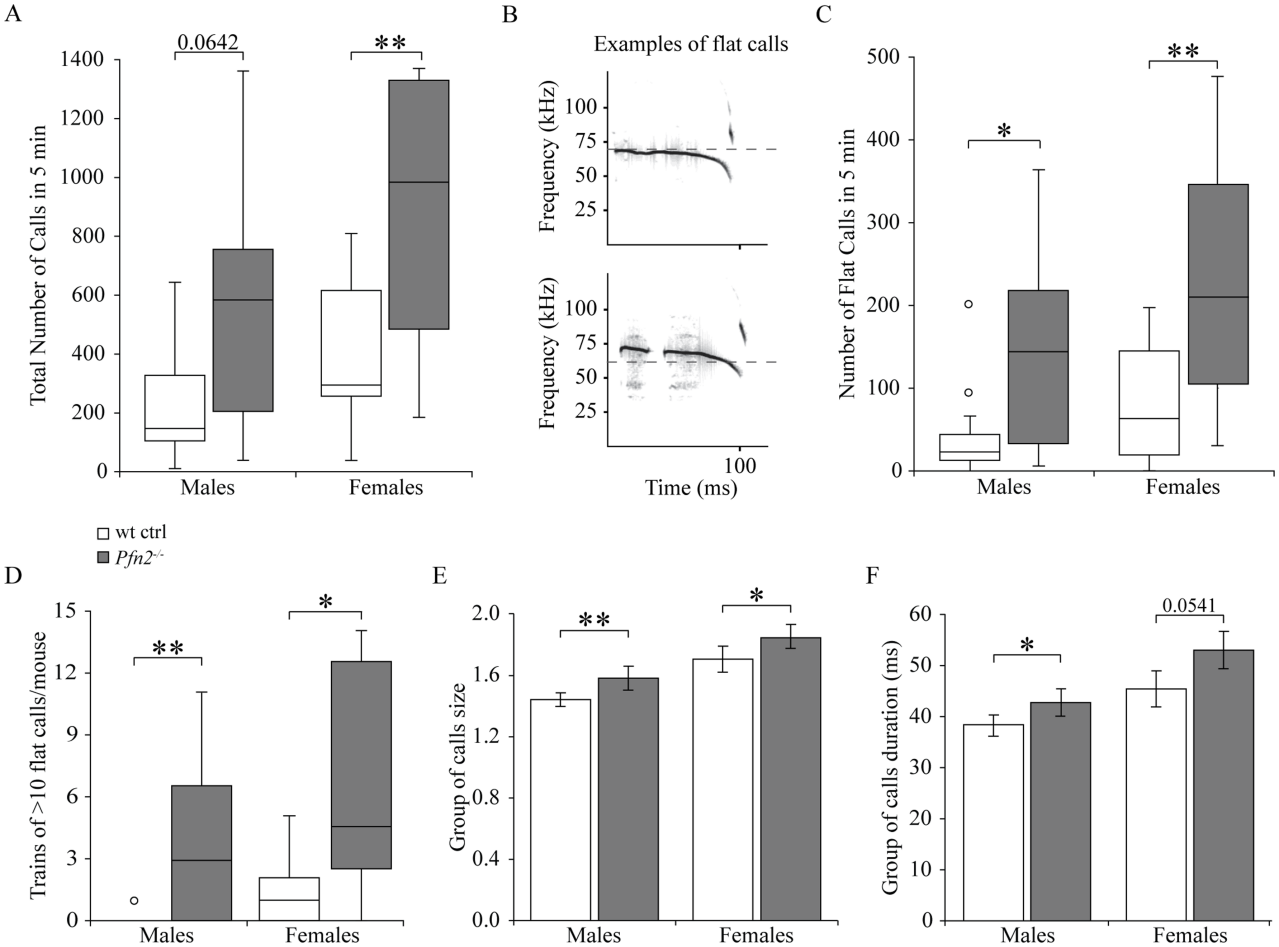
The ultrasonic vocalization pattern in *Pfn2*^−/−^ pups is more monotonous. **(A)** Box plot representing the total number of US calls from P5-P7 *Pfn2*^−/−^ and wt control pups isolated from the nest in 5 min recording time. The median for *Pfn2^−/−^* pups is more than 3-fold the median of wt controls (Mann–Whitney U test, *p* = 0.0642 for males and *p* = 0.0083 for females). **(B)** Graphic representation of two sample flat call traces, one continuous and one interrupted. Of note the uniform frequency below 70 kHz and the length (90–100 ms). **(C)** Box plot representing the number of flat calls from *Pfn2*^−/−^ and wt control pups in 5 min recording time. The median for *Pfn2^−/−^* pups is about 6-fold the median of controls in males and 4-fold in females (Mann–Whitney U test, *p* = 0.0231 for males; *p* = 0.0051 for females). **(D)** The number of trains with more than 10 calls per mouse was strongly increased in *Pfn2* mutants compared to wt controls (Mann–Whitney U test, *p* = 0.0062 for males; *p* = 0.0120 for females). **(E)** The average size of the group of calls (number of shorter calls with inter-call time < 50 ms) was increased in *Pfn2*^−/−^ compared to wt control pups (two-sided *t-*test, *p* = 0.0087 for males; *p* = 0.0298 for females). **(F)** The average duration of group of calls was higher in *Pfn2*^−/−^ compared to wt control pups (two-sided *t-*test, *p* = 0.0351 for males; *p* = 0.0542 for females). *N* = 16 wt ctrl and *N* = 11 *Pfn2*^−/−^ males, *N* = 13 wt ctrl and *N* = 12 *Pfn2*^−/−^ females. *p ≤ 0.05, **p ≤ 0.01.

ASD typically emerge within the first 2 years after birth and crying behavior of babies is the first way of communication in humans. Atypical crying behavior has been reported in incipient ASD infants, often resulting in a negative response of the mother (Esposito and Venuti, 2009). In mouse newborns, up to 8–9 days after birth, “crying” behavior is an important communication pathway, triggered by separation distress. Separated pups emit ultrasonic calls in the 30–110 kHz frequency range to attract their mother’s attention. We monitored ultrasonic vocalizations (USV) from P7 pups and found increased number of USVs in *Pfn2*^−/−^ pups compared to wt controls (Figure 4A, medians *Pfn2*^−/−^ males: 592, wt males: 165; *Pfn2*^−/−^ females: 990, wt females: 311 calls/5 min). Similar observations have been reported in other mouse models of autism (Scattoni et al., 2009; Tsai et al., 2012). Detailed analysis of the USV traces revealed that, despite *Pfn2*^−/−^ and wt control littermates displaying a collection of vocalizations of similar shape and frequency, *Pfn2*^−/−^ pups communicated with a more monotonous vocalization pattern, with persistent use of long (20–90 ms), sometimes interrupted, calls in the middle range of US frequencies (<70–75 kHz) and rather flat frequency modulation (examples shown in Figure 4B). *Pfn2*^−/−^ male and female pups showed, respectively, a 6- and 4-fold median increase of this type of “flat calls” compared to wt control littermates (Figure 4C, medians *Pfn2*^−/−^ males: 144, wt males: 22.5; *Pfn2*^−/−^ females: 216, wt females: 49 flat calls/5 min). In *Pfn2^−/−^* mutants these “flat calls” were often arranged in particularly long trains of calls that were uniformly spaced (with a regular inter-call interval of 150 ± 10 ms, see Supplementary Figure 2) and more intense (<−50 dB, darker gray tone). In comparison to wt control littermates, *Pfn2*^−/−^ male and female pups presented significantly higher number of long trains of calls composed of more than 10 of these monotonous “flat calls” (Figure 4D). About 50% of *Pfn2* mutant pups presented one or more very long trains of over 20 “flat calls,” while none of the control mice showed this behavior (call train example in Supplementary Figure 2). Vocalization patterns are usually made of groups of 2 or 3 closely spaced calls (with short inter-call intervals, <50 ms), within which frequency variations and harmonics are seen. We observed an increased group size and extended group of calls duration in both *Pfn2*^−/−^ males and females compared to wt control littermates (Figures 4E,F).

In summary, *Pfn2^−/−^* pups appeared to be in distress when separated from the mother and engaged in a despair calling behavior with increased calling and reduced frequency modulation (monotony) compared to control pups.

### 2.5 Motor coordination impairment in *Pfn2*^−/−^ mice

**Figure 5:**
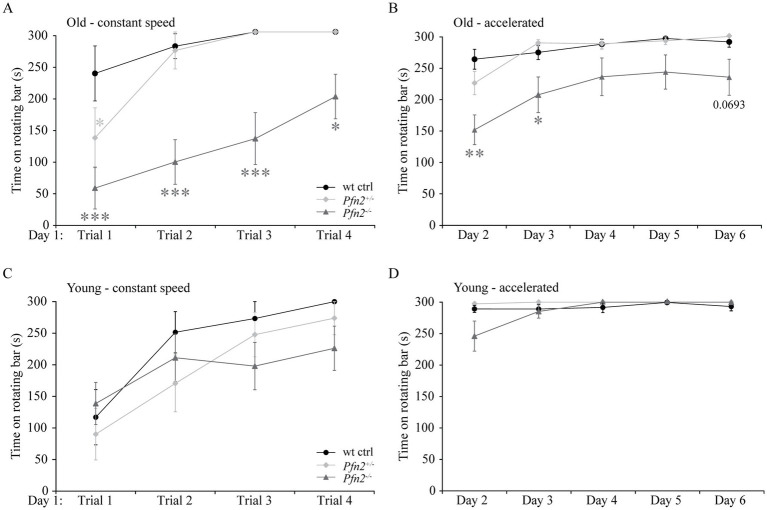
Motor performance decreases with age in *Pfn2*^−/−^ mice. **(A)** Old (6–8 months) *Pfn2*^−/−^ mice showed lower performance on a RotaRod (constant speed, 3 rpm) than wt controls (two-way ANOVA with repeated measures, genotype effect *F*_(2, 25)_ = 19.09, *p* < 0.0001; Tukey’s *post-hoc* test for *Pfn2*^−/−^ vs. wt control: Trial 1, *p* = 0.0001; Trial 2. *p* = 0.0001; Trial 3, *p* = 0.0004; and Trial 4, *p* = 0.0487). *Pfn2*^+/−^ mice showed a motor deficit only in Trial 1 (*p* = 0.0425). **(B)** Old *Pfn2*^−/−^ mice also showed lower performance on a RotaRod in an accelerated rotation paradigm (3–30 rpm in 300 s: two-way ANOVA with repeated measures, genotype effect *F*_(2, 25)_ = 6.285, *p* = 0.0061; Tukey’s *post-hoc* test for *Pfn2*^−/−^ vs. wt control: Day 2, *p* < 0.0001; Day 3, *p* = 0.0224; Day 6, *p* = 0.0693). Notably, motor learning remained unaffected [Day effect *F*_(4, 100)_ = 22.68, *p* < 0.0001]. **(C)** In young mice (3–5 months) no significant differences were seen between *Pfn2*^−/−^, *Pfn2*^+/−^ and wildtype mice in the performance at constant speed, as well as **(D)** in the accelerated Rotarod mode. Represented data are the average of the best performances of the day ± SEM. Old mice, *N* = 9 wt ctrl and *Pfn2*^−/−^, *N* = 10 *Pfn2*^+/−^; young mice *N* = 11 per genotype. *p ≤ 0.05, **p ≤ 0.01, ***p ≤ 0.001.

In addition to the core symptoms and the possible communication deficits, impaired motor coordination is often found as a comorbidity in ASD (Fournier et al., 2010). We tested basic motor performance and coordination in *Pfn2^−/−^* mice using a fixed rotation speed RotaRod paradigm, while we assessed motor learning by constantly increasing the rotation speed. In older *Pfn2* mutants, motor performance and coordination at fixed low rotation speed was significantly impaired (Figures 5A,B), while younger mutant mice did not show significant deficits (Figures 5C,D). Motor learning, on the other hand, was unaffected at all ages in *Pfn2^−/−^* mice, in line with previous findings in the conditioned learning paradigm (Pilo Boyl et al., 2007). Thus, *Pfn2*^−/−^ mice showed an age dependent impairment of motor performance that could depend on impaired coordination.

**Figure 6:**
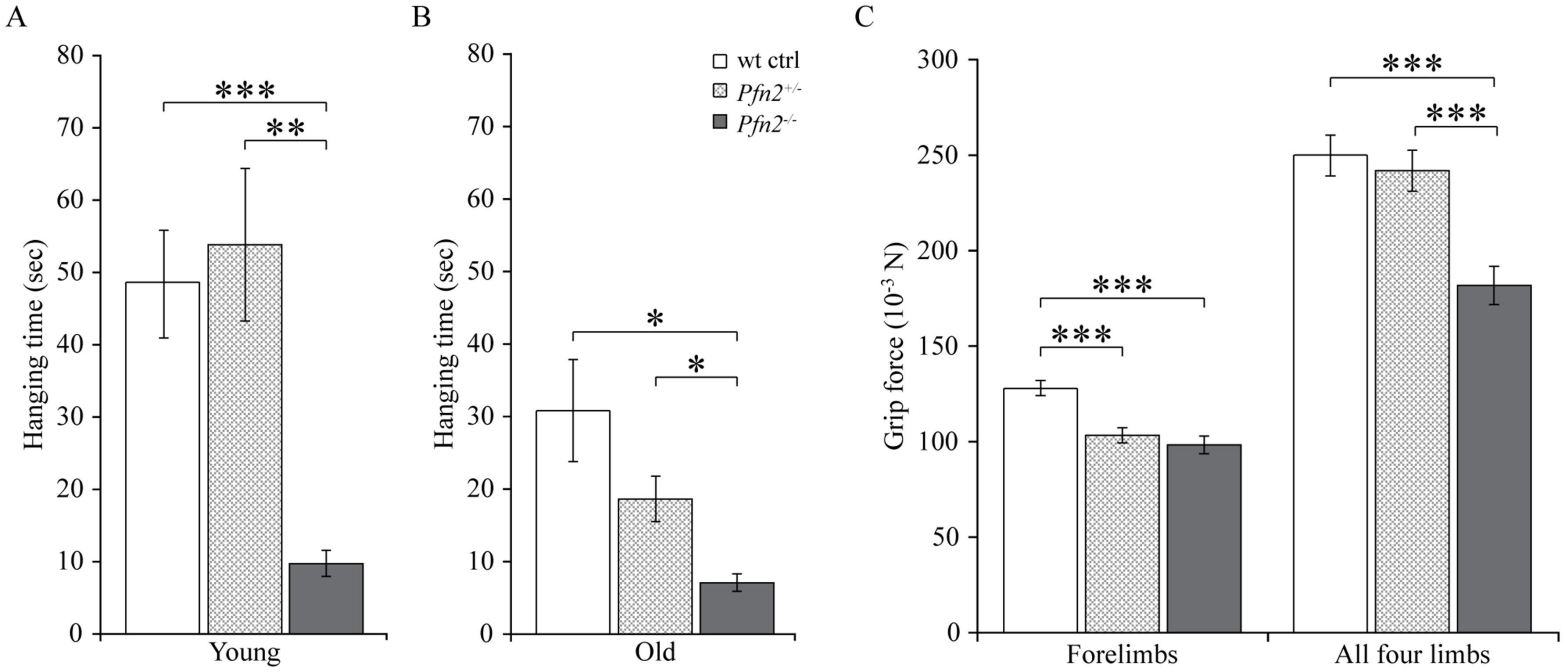
Motor coordination is reduced in *Pfn2*^−/−^ mice. **(A)** In the Hanging test young (3–5 months) *Pfn2*^−/−^ mice showed deficits compared to wt controls (two-sided *t-*test, *p* = 0.0004) and *Pfn2*^+/−^ mice (two-sided *t-*test, *p* = 0.0018). **(B)** Older mice (6–8 months) performed in general less well than young mice, but *Pfn2*^−/−^ mutants again displayed significant coordination impairment compared to wt controls (two-sided *t-*test, *p* = 0.0103) and to *Pfn2*^+/−^ mice (two-sided *t-*test, *p* = 0.0207). Heterozygous mice showed an age dependent loss of coordination ability. **(C)** Independently of the mouse age, muscle strength of the forelimbs was significantly reduced in both *Pfn2*^−/−^ mice (two-sided *t-*test, *p* < 0.0001) and *Pfn2*^+/−^ mice (two-sided *t-*test, *p* < 0.0001) compared to wt controls. When all 4 limbs were engaged, only *Pfn2*^−/−^ mice were found impaired (two-sided *t-*test, *p* = 0.0003), while *Pfn2*^+/−^ mice performed similarly to wt controls (two-sided *t-*test, *p* = 0.0002 *Pfn2*^+/−^ vs. *Pfn2*^−/−^ mice). Same animals as in Figure 5 were used. *p ≤ 0.05, **p ≤ 0.01, ***p ≤ 0.001.

Coordination skills were further investigated with the Hanging test, which addresses both muscle strength and coordination between legs and body. The performance of *Pfn2^−/−^* mice was markedly impaired at both young (Figure 6A) and older age (Figure 6B). In the group of older mice, the *Pfn2*^+/−^ animals showed a gene dosage-dependent intermediate phenotype.

To discriminate between muscle strength and coordination, we measured the mice grip strength. While gripping only with the forelimbs depends exclusively on the muscle control and strength, when the mice are allowed to grip with all four limbs, the coordination between fore- and hindlimbs affects the gripping strength. Both *Pfn2*^−/−^ and *Pfn2*^+/−^ mice showed about 20% less pulling strength with their forelimbs (Figure 6C, left), suggesting a mild deficit in muscle strength. However, grip strength with all four limbs was reduced only in *Pfn2^−/−^* mice (Figure 6C, right), while *Pfn2*^+/−^ animals were comparable to wt controls, suggesting that the main motor function affected by loss of PFN2 was coordination.

Coordination issues could be spotted already at very early age (i.e., at P10) in *Pfn2^−/−^* mice by the characteristic hindlimb clasping when gently lifted by the tail instead of the normal hindlimb outward splaying (Supplementary Figure 3A), a phenotype typically present in mouse models with motor coordination and/or ataxia issues and cerebellar dysfunction (Lalonde and Strazielle, 2011; Yerger et al., 2022). Interestingly, the gait quality was only mildly affected in a foot-printing assay, where we only observed a slightly shorter pace (Supplementary Figure 3B).

Since motor coordination largely depends on cerebellar function, we studied Purkinje cell physiology in *Pfn2^−/−^* mice.

### 2.6 Increased glutamatergic input in Purkinje cells of *Pfn2*^−/−^ mice

Purkinje cells (PC) are large inhibitory neurons of the cerebellar cortex organized in a monolayer between the inner granule cells and the external molecular layer, in which they extend their large dendritic arbors. These arbors receive extensive excitatory input from parallel and climbing fibers that generate dense trains, respectively, of simple and complex spikes (Sugimori and Llinás, 1990; Ghez and Thach, 2010). The central part of the cerebellum comprising the vermis region, the spinocerebellum, has been shown to regulate motor execution (Ghez and Thach, 2010).

**Figure 7:**
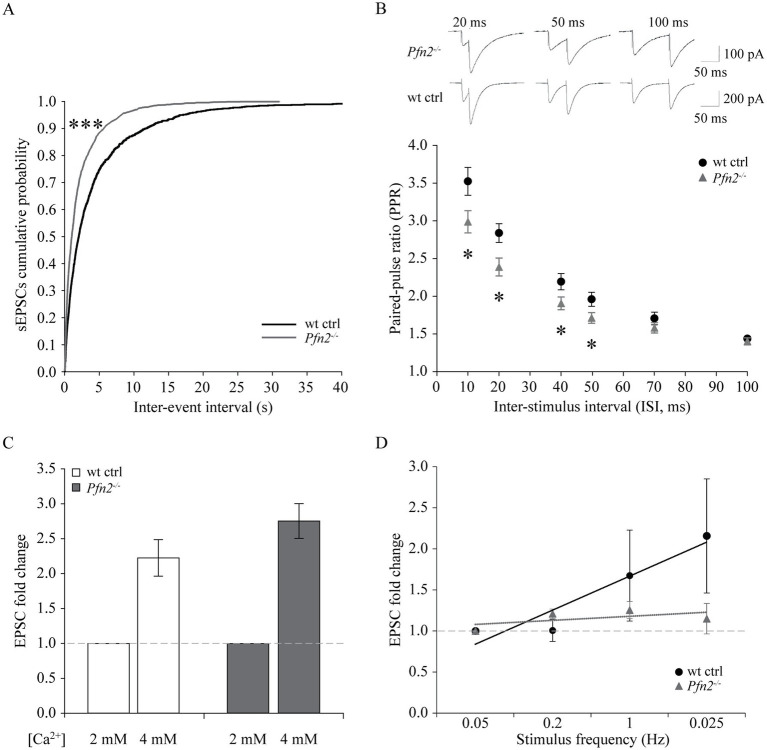
Increased glutamatergic input into Purkinje cells of *Pfn2*^−/−^ mice. **(A)** Percentage cumulative frequency graphs of the average inter-event interval (IEI) distribution of sEPSCs recorded in PCs. The leftward shift for *Pfn2*^−/−^ neurons indicates higher percentage of small IEIs, equivalent to increased frequency of events (Kolmogorov–Smirnov test, *p* < 0.001). *N/n* = 2/4 wt ctrl, *N/n* = 3/6 *Pfn2*^−/−^ mice/cells. **(B)** Paired-pulse ratio (PPR) of parallel fiber-Purkinje cell synapses at different inter-stimulus intervals (ISI: 10, 20, 40, 50, 70, 100 ms). At ISI ≤ 50 ms, *Pfn2*^−/−^ neurons showed significantly reduced PPR compared to wt control, indicating higher probability of synaptic vesicle exocytosis. Upper traces: sample traces of *Pfn2*^−/−^ and wt control neurons at different ISI. *N/n* = 3/8 wt ctrl and *N/n* = 4/10 *Pfn2*^−/−^ mice/cells. **(C)** Hyper-excitability of *Pfn2*^−/−^ neurons in response to extracellular calcium increase. Raising the [Ca^2+^] from 2 to 4 mM increased the EPSCs by 2.75 ± 0.25-fold in *Pfn2*^−/−^ neurons, but only by 2.22 ± 0.26-fold in wt control neurons. Dotted line marks the normalized value for [Ca^2+^] = 2 mM. *N/n* = 2/6 wt ctrl and *N/n* = 2/6 *Pfn2*^−/−^ mice/cells. **(D)** Normalized frequency-dependent plasticity of evoked EPSCs increasing the stimulation from 0.05 Hz to 1 Hz. Stimulation was then reduced to 0.025 Hz to test the maintenance of the potentiation. *Pfn2*^−/−^ neurons showed potentiation of EPSCs at 0.2 Hz stimulation but immediately reached a low plateau, while wt control neurons further potentiated their response up to 1 Hz stimulation and maintained it during the final low Hz stimulation. Linear regression analysis shows the different slope of the wt and *Pfn2*^−/−^ neurons facilitation (respectively, black and grey dotted lines). The dashed grey line indicates the normalized level at the initial 0.05 Hz stimulation. *N/n* = 2/3 wt ctrl and *N/n* = 2/3 *Pfn2*^−/−^ mice/cells. *p ≤ 0.05, ***p ≤ 0.001.

We performed electrophysiological studies on Purkinje cells. Spontaneous excitatory post-synaptic currents (sEPSCs) recorded from PCs showed a significant leftward shift of the cumulative probability of the inter-event intervals in *Pfn2*^−/−^ mice, indicating a significant increase of the sEPSCs frequency (Figure 7A), while their amplitude distribution did not change significantly (not shown). Moreover, the paired-pulse ratio (PPR) between pairs of stimuli on parallel fibers with increasing inter-stimulus interval (ISI) was significantly reduced in *Pfn2*^−/−^ cells between 10 and 50 ms paired-pulse (PP) intervals (Figure 7B), indicative of an altered short-term synaptic plasticity that we can attribute to higher release probability of glutamatergic vesicles, since we have shown in a previous work that synaptic density is not altered in *Pfn2*^−/−^ mice (Pilo Boyl et al., 2007). To confirm the increased excitatory input from parallel fibers onto PCs, two additional experiments were performed. First, we studied the effect of PFN2 depletion when manipulating the release probability by increasing the extracellular [Ca^2+^] from 2 mM, in standard control ACSF conditions, to 4 mM. In these conditions, the increase of EPSCs amplitude was 22% larger in *Pfn2^−/−^* than wt neurons (Figure 7C, *Pfn2*^−/−^: 2.75 ± 0.25 vs. wt: 2.27 ± 0.24-fold increase), indicating that PCs in *Pfn2^−/−^* animals experience higher excitatory stimulation. In a second experiment, frequency-dependent plasticity of the parallel fiber-PC synapse was analyzed. Increasing the frequency of stimulation from 0.05 to 0.2 Hz produced a mild potentiation of the EPSCs in both *Pfn2*^−/−^ and wt control cells (Figure 7D), however, an additional frequency increase up to 1 Hz produced potentiation only in wt control neurons but not in *Pfn2*^−/−^ cells that reached a plateau at much lower stimulation frequency than wt controls (Figure 7D, linear regressions, dotted lines). These results support the previous findings of a higher basal probability of glutamate release in *Pfn2*^−/−^ glutamatergic neurons.

In summary, our results showed that PFN2 is regulating cerebellar physiology by restraining the glutamatergic input in PCs from afferent projections. The findings in the cerebellum are in line with our previous observations in striatum (Pilo Boyl et al., 2007), strengthening the point that PFN2 has a critical function in limiting glutamatergic release in the CNS.

### 2.7 Age-dependent loss of Purkinje cells in *Pfn2*^−/−^ mice

**Figure 8:**
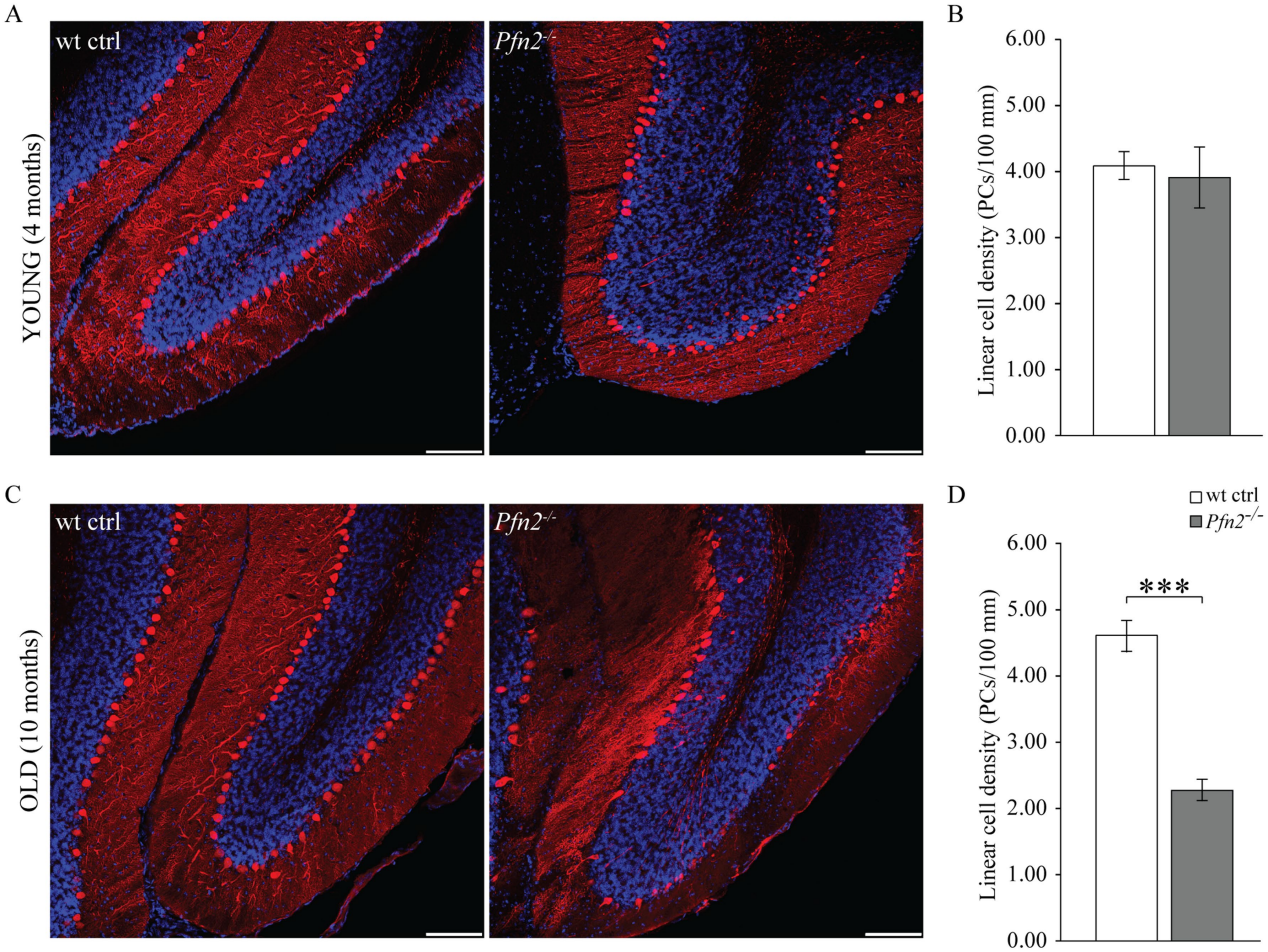
Age dependent loss of Purkinje cells in *Pfn2*^−/−^ mice. **(A)** Sample confocal microscopy images of cerebellar folia from 4 months old wt control (left) and *Pfn2*^−/−^ (right) mice, using calbindin antibodies (red) to specifically identify Purkinje cells and DAPI (blue) for the nuclei to highlight the granule cell layer. **(B)** Quantification of the linear cell density of Purkinje cells in 4 months old mice: no difference between *Pfn2*^−/−^ and wt ctrl mice was found. **(C)** Sample confocal microscopy images of cerebellar folia from 10 months old wt control (left) and *Pfn2*^−/−^ (right) mice. Large patches of PCs were lost in *Pfn2*^−/−^ compared to wt control mice. **(D)** Quantification of the linear cell density of Purkinje cells in 8–10 months old mice: significantly reduced density in *Pfn2*^−/−^ mice compared to controls (two-sided *t-*test, *p* = 2.6*10^−8^). Scale bar 100 μm. Quantification of *N* = 3 wt ctrl and *N* = 3 *Pfn2*^−/−^ 4 months old and 8–10 months old mice, from at least 2 stained sagittal spinocerebellar slices per mouse. ***p ≤ 0.001.

PCs of the cerebellum are a class of neurons particularly vulnerable to excessive glutamatergic input due to their double connectivity with parallel and climbing fibers. Excitotoxicity is predominantly a glutamate-dependent event, mediated by NMDA receptors, which, in conditions of dysregulated glutamate homeostasis, cause excessive and/or prolonged rises in intracellular calcium that trigger neuronal cell death pathways (for a review, see Armada-Moreira et al., 2020). The increased glutamatergic output observed in *profilin 2* knock-out mice led us to investigate the structure of the PC layer in 4 and 10 months old *Pfn2*^−/−^ mice in spinocerebellar slices, the time points aligning with the motor coordination experiments previously described. At 4 months the PC layer in *Pfn2*^−/−^ mice was essentially normal (Figure 8A), showing no significant difference in the linear density of PCs compared to wt controls (Figure 8B). At this age motor performance was not affected. However, at 10 months of age we observed severe changes in *Pfn2* mutant mice: the PC layer in the central part of the cerebellum was highly disorganized, with long stretches completely devoid of PC cell bodies (Figure 8C), resulting in significantly reduced linear PC density (Figure 8D, *Pfn2*^−/−^: 2.27 ± 0.23 vs. wt: 4.60 ± 0.17 cells/100 μm). Severe motor coordination and performance deficits were indeed present at this age. These findings also suggest that PCs in *Pfn2*^−/−^ mice are slowly lost throughout life.

Interestingly, we also observed in *Pfn2^−/−^* cerebella the presence of calbindin positive cells in the internal granular layer (Figures 8A,C right panels), which hint to a late neurodevelopmental migration defect, conceivable according to the proven function of profilins in cell motility (Zhou et al., 2019).

### 2.8 Imbalanced glutamatergic/GABAergic transmission in the hippocampus of *Pfn2*^−/−^ mice

**Figure 9:**
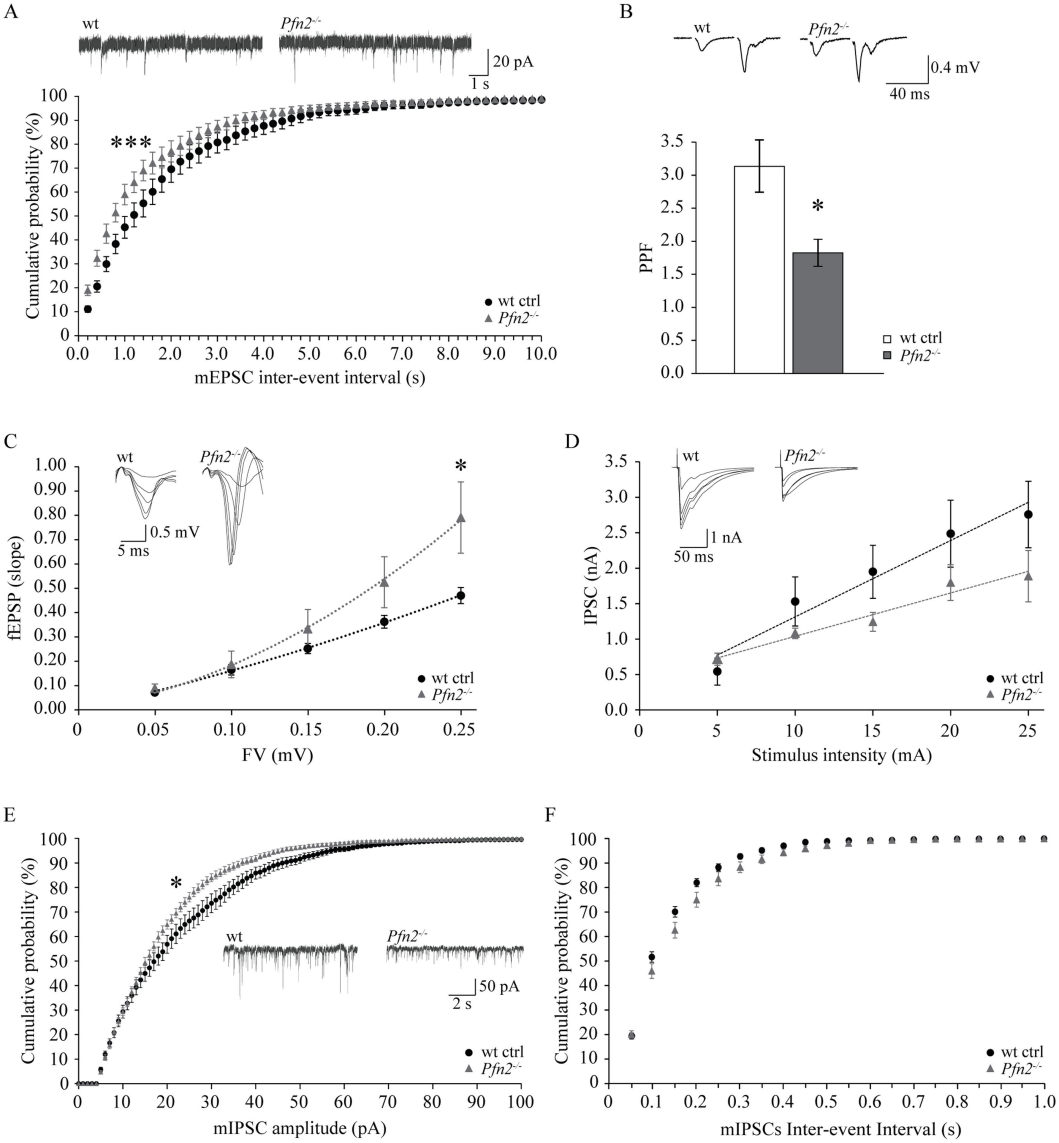
Increased ratio of excitatory versus inhibitory synaptic inputs in the hippocampus of *Pfn2^−/−^* mice. **(A)** Percentage cumulative frequency plot of the inter-event intervals distributions of mEPSCs recorded in CA3 neurons showed increased frequency of events in *Pfn2^−/−^* compared to wt control mice (Kolmogorov–Smirnov *p* = 0.001). Upper traces, sample mEPSCs traces. *N/n* = 4/7 wt ctrl and *N/n* = 3/8 *Pfn2^−/−^* mice/cells. **(B)** Field PPR with 40 ms inter-stimulus interval in the Schaffer collaterals of *Pfn2^−/−^* mice was significantly reduced by ca. 40% compared to wt controls (two-sided *t-*test *p* = 0.0102), indicating higher vesicle exocytosis probability. Upper traces: sample fEPSC response traces with 40 ms ISI. *N/n* = 6/15 wt ctrl and *N/n* = 5/9 *Pfn2^−/−^* mice/slices. **(C)** fEPSP input–output (I-O) relation in the Schaffer collaterals was increased in *Pfn2^−/−^* mice compared to wt controls, indicating a stronger excitatory transmission. Polynomial regression analysis (dotted lines) shows the higher I-O relation in *Pfn2^−/−^* slices compared to wt controls (y = 8.6734x^2^ + 0.9515x for *Pfn2^−/−^* mice, y = 1.9042x^2^ + 1.4053x for wt mice). Upper traces: superimposed traces from a sample recording at increasing fiber volley. *N/n* = 7/19 ctrl and *N/n* = 7/14 *Pfn2^−/−^* mice/slices. **(D)** IPSC I-O relation showed a mild decrease of the inhibitory synaptic transmission in *Pfn2^−/−^* pyramidal neurons compared to wt controls. Upper traces: superimposed traces from a sample recording at increasing stimulus intensity. *N/n* = 3/6 wt and *N/n* = 3/5 *Pfn2^−/−^* mice/cells. **(E)** Percentage cumulative frequency plot of the amplitudes of mIPSCs shows a significant decrease of inhibitory transmission in *Pfn2^−/−^* mice compared to wt littermate controls (Kolmogorov–Smirnov, *p* = 0.025). Inset traces: sample mIPSCs traces. **(F)** No difference was found in the inter-event interval of mIPSCs between *Pfn2^−/−^* and wt control mice, as shown by the cumulative probability plot. *N/n* = 3/10 wt and *N/n* = 4/10 *Pfn2^−/−^* mice/cells. *p ≤ 0.05, ***p ≤ 0.001.

The etiology of ASD remains largely unknown, but one hypothesis suggests it could be based on a shift of the excitation/inhibition (E/I) ratio in the brain or in select neuronal circuits (Rubenstein and Merzenich, 2003; Lee et al., 2017). We previously documented increased glutamatergic transmission in *Pfn2^−/−^* mice, through higher glutamatergic vesicle exocytosis (Pilo Boyl et al., 2007), and we provided additional findings in this work supporting the possibility that a general increase of the glutamatergic neurotransmission not counterbalanced by an increase of GABAergic inhibition could underlie the autistic-like traits of *Pfn2^−/−^* mice. In order to prove this, inhibition needs to be measured. The CA3-CA1 Schaffer collaterals in the hippocampus are a well-characterized brain circuit to address the balance between excitatory and inhibitory synaptic inputs. We first analyzed excitatory transmission in this circuit using three different paradigms. We recorded miniature excitatory post-synaptic currents (mEPSCs) from CA1 pyramidal neurons, and then, while blocking GABA_A_ receptors, we measured the paired-pulse ratio (PPR) and calculated the input–output (I-O) relation in the Schaffer collaterals pathway. Glutamatergic transmission, similarly to all other previously tested circuits, was increased also in the hippocampus of *Pfn2^−/−^* mice: mEPSCs were significantly more frequent in *Pfn2^−/−^* mice compared to wt controls, as indicated by the leftward shift of the cumulative curve for the inter-event intervals (Figure 9A), with only a mild reduction in their amplitude (Supplementary Figure 4A). By means of extracellular field recordings we showed that the paired-pulse ratio at 40 ms ISI was reduced by 40% (Figure 9B, *Pfn2^−/−^* 3.08 ± 0.30 vs. wt 1.80 ± 0.18) while the I-O relation was significantly increased in *Pfn2^−/−^* mice compared to control littermates (Figure 9C), indicating that for a similar number of stimulated pre-synaptic fibers, *Pfn2*^−/−^ mice exhibited an increased post-synaptic response. There was no difference in the stimulation intensity to obtain the same fiber volley in the two genotypes (Supplementary Figure 4B), therefore similar numbers of pre-synaptic fibers responded to the same stimulation level.

Next, we studied inhibitory transmission in the same circuit, measuring miniature inhibitory post-synaptic currents (mIPSCs) in CA1 pyramidal neurons and calculating the I-O relation while blocking AMPA receptors. Contrary to the excitatory drive that was clearly increased, the inhibitory transmission was unchanged or mildly reduced. In the input–output relation experiment, *Pfn2^−/−^* pyramidal neurons showed a tendential loss of GABA-dependent currents (Figure 9D), which was confirmed by a significant decrease in the amplitudes of mIPSCs (Figure 9E) with no change in mIPSCs frequencies (Figure 9F), suggesting a prevalent post-synaptic defect in CA1 pyramidal neurons, where PFN2 was shown to interact with gephyrin scaffolds and was proposed to regulate receptor densities (Giesemann et al., 2003; Murk et al., 2012). Overall our data indicate that in *Pfn2^−/−^* mice excitatory synaptic inputs are increased while inhibitory synaptic inputs are reduced, thereby suggesting a shift of the E/I balance toward excessive excitation.

## 3 Discussion

In this work we report three important findings based on the constitutive *Pfn2* knock-out mouse model: (1) that lack of PFN2 causes a variety of autistic-like phenotypes; (2) that increased glutamate release in *Pfn2^−/−^* mice alters excitatory synaptic connectivity in multiple circuits, suggesting that this is a general defect of all glutamatergic synapses lacking PFN2; (3) that inhibitory neurotransmission is not upregulated to counterbalance the increased glutamatergic drive in *Pfn2^−/−^* mice, thus suggesting a net increase of the excitation/inhibition ratio.

The conventional knock-out of *Pfn2* produces conspicuous deficits in maternal/paternal behavior, vocal communication, and motor coordination, with an age-dependent effect on motor performance that correlates with the loss of Purkinje cells in the cerebellum. Therefore, the phenotypic spectrum ranges from core symptoms to common comorbidities of ASD, including epileptic seizures, measured in about 12.5% of the mutant mice. In recent years, whole-exome sequencing (WES) studies of ever larger cohorts of autism patients have uncovered a significant number of genes causative of ASD, which appear to be involved in a wide spectrum of cellular functions (De Rubeis et al., 2014; Iossifov et al., 2014; Satterstrom et al., 2020). Nevertheless, many more genes are thought to be involved, with variable degree of impact on autism risk, according to estimations based on statistical simulations and the development of more powerful statistical tools to analyze the mutations discovered by WES, which integrate polygenicity and protein interactions networks (Neale et al., 2012; Sanders et al., 2012; He et al., 2013; De Rubeis et al., 2014). Consensus is emerging that the ultimate physiological target of the mutations is either synaptic function or brain circuit development, in particular through cortical neurons specification (Baudouin, 2014; Fromer et al., 2014; Satterstrom et al., 2020). The human gene *PROFILIN 2* has not yet appeared in WES studies of genomes of ASD patients. However, current knowledge on PFN2 function in brain, as well as the data presented in this work, would support its standing as a risk gene for neurological disorders, including ASD. In the mouse, PFN2 function appears to be mainly restricted to regulating synaptic activity (Ackermann and Matus, 2003; Gareus et al., 2006; Pilo Boyl et al., 2007) and to maintaining dendritic arborization (Michaelsen et al., 2010; Di Domenico et al., 2021), thus clearly taking part in the shaping of neuronal connectivity. Moreover, profilin 2 has been shown to interact with a number of ligands and to participate in signaling pathways that have been implicated in ASD. For example, the small RHO GTPases regulate actin dynamics in developing neurons and indeed have been implicated as additional ASD risk genes in models that cluster genes strongly associated to and co-expressed with discovered ASD causing genes in key tissues, such as mid-fetal pre-frontal and somato-sensory cortex (De Rubeis et al., 2014). The RHOA pathway, involving the downstream effector kinase ROCK (RHO-associated coiled-coil containing kinase), regulates in very specific ways dendrite sprouting, extension, and ramification, as well as axon branching during early and late neuronal development (Bito et al., 2000; Nakayama et al., 2000; Ohnami et al., 2008) and can therefore affect the establishment of correct neuronal connectivity. ROCK kinase has been shown to specifically interact with PFN2 (Witke et al., 1998; Da Silva et al., 2003) and the RHOA/ROCK pathway to act through phosphorylation of PFN2 to limit early neurite sprouting of cultured mouse hippocampal neurons (Da Silva et al., 2003). On the other hand, the RAC1 pathway functions by activating the WAVE regulatory complex (WRC), a molecular machine that promotes actin nucleation to form branched actin networks (Pollard, 2007) essential for axonal branching and growth-cone dynamics (Spillane et al., 2011; Miguel-Ruiz and Letourneau, 2014), dendritic branching (Stürner et al., 2019), as well as spine sprouting, structure, and potentiation (Korobova and Svitkina, 2010). CYFIP1 and NCKAP1 that are part of the WRC, are both established ASD risk genes. PFN2 is found in complexes with the two CYFIP paralogues (Witke et al., 1998) and ultimately takes part in the actin polymerization process initiated by the WRC, recruited as profilactin moiety, a 1:1 PFN2-G-actin complex. Thus, copy number variation (CNV) or single nucleotide variation (SNV) in the *PFN2* gene could well contribute through numerous pathways to alterations in neuronal development and physiology, ultimately leading to ASD.

In view of the fact that *Pfn2* is highly expressed in neurons across the entire brain (Witke et al., 1998; Da Silva et al., 2003; Pilo Boyl et al., 2007), that PFN2 is involved in pathways connected to ASD risk or ASD causing genes, as just described, and that PFN2 is mainly functioning in excitatory synaptic physiology and in a more limited way in inhibitory synaptic physiology, as shown in this work, we believe that it is not so surprising that the *Pfn2* knock-out mouse reproduces a variety of autistic-like traits. In the *Pfn2* knock-out mouse model the loss of maternal (and paternal) behavior was remarkably strong, while in the sociability test the deficits were less evident, most likely due to the confounding factor of the previously reported novelty-induced hyper-excitability behavior (Pilo Boyl et al., 2007). The hyper-excitability phenotype possibly also masked repetitive behavior and resistance to changes, which did not appear particularly strong in the applied behavioral paradigms, while the communication behavior of *Pfn2* mutant mouse pups was significantly altered, with a pattern reminiscent of infant vocalization in ASD (Esposito and Venuti, 2009). We acknowledge that not all standard autistic-like behaviors are present or are markedly detectable in the mouse model, but it is not unusual that behavioral characterization of mouse models reveals heterogeneity in the manifestation of the autistic-like phenotypes, with some that appear stronger and others evidently mild, even among the core symptoms. The overall picture obtained for the *Pfn2* ko mouse model is not uncommon when characterizing animal models of human diseases, and in particular ASD, for at least two reasons: first, animal models often exhibit only a part of the full spectrum of phenotypes seen in polygenic human diseases like autism (Neale et al., 2012; McCarroll and Hyman, 2013) because the animal models typically carry the mutation of only one gene, as in the case of the *Pfn2* knockout mouse; secondly, one has to consider that inbred mice have a relatively narrow genetic background variation, therefore if in a colony the genetic background is tolerant of a specific genetic mutation for a certain behavior, there is little chance that one can find individual mice with stronger or diverse phenotypes for that behavior. As for many neurological disorders, it is unlikely that *PFN2* mutations would occur homozygously in humans, however the complete *Pfn2* knock-out mouse model employed in this work facilitates to discern which aspects of neuronal development, physiology, and behavior are critically affected when the gene is inactivated. Moreover, a homozygous knock-out approach in mouse helps to overcome the higher robustness of the mouse CNS compared to the more complex and therefore vulnerable human CNS in the study of these phenotypes. It is anyway remarkable that even *Pfn2* heterozygous mice show intermediate phenotypes in some of the tested behaviors, strengthening the expectation of discovering CNVs or SNVs in *PFN2* in humans.

Concerning the excess glutamate release in *Pfn2^−/−^* mice, we have previously reported the mechanism by which PFN2 regulates excitatory pre-synaptic terminal physiology, with loss of PFN2 increasing pre-synaptic vesicle release from excitatory synapses in the cortico-striatal pathway and therefore enhancing circuit excitation (Pilo Boyl et al., 2007). With this work we extend the study, showing that the mechanism found originally in one circuit is reasonably a general mechanism for glutamatergic neurons, since also the parallel fiber signaling in the cerebellum and the Schaffer collaterals system in the hippocampus are affected in a comparable way when PFN2 is missing, and they are very different circuits with only in common the released neurotransmitter, glutamate. Therefore, one should expect an overall increase of glutamatergic excitation over the entire central nervous system when PFN2 is below threshold. A consequence of the increased glutamatergic neurotransmission in the absence of PFN2 is the progressive loss of cerebellar Purkinje cells that we observed in *Pfn2* ko mice. PCs are the most susceptible cells of the brain to excitotoxicity, due to their double innervation from glutamatergic climbing and parallel fibers (Ghez and Thach, 2010). The excitotoxicity hypothesis is supported by the fact that PC loss is progressive and starts after a few months of the mouse life, excluding a major neurodevelopmental defect. The progressive loss of PCs is also paralleled at the behavioral level by the age-dependent deterioration of the coordination phenotype, which is in line with the involvement of the Purkinje cells of the spinocerebellar region to regulate limb motor functions (Ghez and Thach, 2010). Purkinje cell loss has been found in a subset of autism patients following post-mortem analysis of their brains (Skefos et al., 2014). Unfortunately, no connection was made with the type of genetic or physiological dysfunction that caused the autism phenotype in these individuals, in particular if it could be an increased excitation/inhibition ratio.

Lastly, in this work we add an important notion to the understanding of the loss of PFN2 in the brain. When PFN2 is absent, inhibitory GABAergic neurotransmission is not affected in the same way as the excitatory, being on the contrary unaltered or post-synaptically mildly depressed in the same glutamatergic neurons that receive higher glutamatergic excitatory inputs from the CA3 region of the hippocampus. It has been since long reported the interaction of PFN2 with the inhibitory post-synaptic scaffold protein gephyrin (Giesemann et al., 2003; Murk et al., 2012), but to the best of our knowledge no follow up studies have been conducted to explore the physiological significance of this interaction. We report here for the first time one physiological outcome of disrupting the interaction between PFN2 and gephyrin: the decrease of inhibitory currents in CA1 pyramidal neurons. This reduction of post-synaptic inhibitory currents could be due to decreased clustering of GABA receptors or to their faulty assembly or function. The consequence of this finding is that the net effect of depleting PFN2 in the brain is, therefore, a likely increase of the excitation/inhibition ratio, mainly due to an increase of glutamatergic neurotransmission in the brain. The E/I imbalance has been proposed as one possible cause of ASD (Rubenstein and Merzenich, 2003), and has been already demonstrated, as proof-of-principle, by the knock-out mouse model of the *eIF4E binding protein 2* (*Eif4epb2*) (Gkogkas et al., 2013), which showed increase in excitatory transmission, and by the knock-out of *GABAA receptor beta 3 subunit* (*Gabrb3*), which had decreased inhibitory function (DeLorey et al., 1998). The *Pfn2* knock-out mouse appears to be in line with this mechanism and these other mouse models, with a particular phenotypic agreement to the first model. One probable consequence of the increase of the E/I ratio at the circuit level is the epileptic seizures phenotype that we observed in the *Pfn2* ko mouse. Tonic and tonic–clonic seizures induced by sensory stimulation were found in about 12.5% of the mutant mice, starting from young to middle age, but measurement of epileptic activity in freely moving *Pfn2* mutant mice might reveal a higher prevalence. Epilepsy is a comorbidity observed in a subset of ASD patients. Recent meta-analyses of studies on autistic patients have shown that in humans epilepsy has an average prevalence of 15–16%, although with higher pooled prevalence in subjects with intellectual disability (21–24%) than in those without (8–9%), and that this prevalence increases with age (Amiet et al., 2008; Woolfenden et al., 2012). Our findings in the *Pfn2* ko mouse model appear mostly in line with these data. Unfortunately, nothing is known yet about the causes of epilepsy as a comorbidity of ASD in humans. Our study, as well as others (DeLorey et al., 1998; Cobos et al., 2005), suggest that one possible cause of the epileptic comorbidity might reside in synaptic dysfunctions that raise the E/I ratio.

In conclusion, the *Pfn2* knock-out mouse could provide a model to study therapeutic interventions for a subset of autistic disorders dependent on increased excitation/inhibition ratio in the nervous system. According to the physiological function of PFN2 in neurons and its protein–protein interactions network, it is not unlikely that in future WES studies with even larger patients’ cohorts and more complex statistical analysis methods *PFN2* might be detected as an ASD risk gene. The knock-out mouse phenotype strongly supports this possibility, since several ASD symptoms, both core and comorbid, are reproduced and are similar to those in other ASD mouse models.

## 4 Materials and methods

### 4.1 Mice

The *Pfn2* knock-out (*Pfn2^−/−^*) mouse model was previously described (Pilo Boyl et al., 2007). All mice used in the experiments were littermates generated by crossing heterozygous parents. Mice were socially housed with a standard 12 h light/dark cycle at 22 °C and 50–55% humidity, with free access to water and food pellets. All experiments were performed according to EU regulations (Licenses n. 19/2005-B and AZ 84–02.04.2013.A233).

### 4.2 Behavior

Male mice 3–5-months old (“young”) were used in behavioral experiments, except where differently indicated. Mice indicated as “old” were aged 6–10 months. Maternal behavior: pregnant females were single-housed a week before delivery. Litter survival was assessed 10 days post-partum (P10). In a second approach, pregnant female and male were left together to allow cross-fostering. Maternal behavior, pup retrieval: P5 pups from *Pfn2*^−/−^ and wt control females were dispersed in the cage and time for retrieval of the first pup and of the entire litter was scored up to 30 min. Social interaction: the test chamber was built according to Moy et al. (2007). The experiment consisted of two 10 min trials: first, the test animal explored a tripartite chamber containing two empty cages in the outer compartments; second, the same test animal was confronted with one empty cage and one cage containing the stranger mouse, with a modification of the original test to ensure a non-aversive context for *Pfn2^−/−^* mice that are hyper-excitable (Pilo Boyl et al., 2007): we used juvenile mice of 3.5 weeks (P25) of age as social partners. Stereotypic and repetitive behavior: self-grooming, digging, jerking, circling, and wall-leaning behavior was scored as number of events in 300 s after transfer into a novel cage. Y-maze: mice were allowed to freely explore the Y maze for 5 min and percent (%) of SPA (spontaneous alternation) and SAR (same arm return) was calculated with respect to total arm entries. Ultrasound vocalizations (USV): USVs were measured in P5-P9 male and female pups (Branchi et al., 2001). Pups were singularly taken from the mother and placed in a soundproof chamber at room temperature (22 °C) under a condenser ultrasound microphone (Avisoft Bioacoustics CM16/CMPA) connected to an UltraSoundGate 116Hb and data were collected for 300 s with the Avisoft Recorder 2.76 in 16-bit format. Analysis of USVs was performed using the Avisoft SASLab Pro software (Avisoft Bioacoustics, Berlin DE). The low frequency (<70–75 kHz) long calls (>20 ms) and the call trains (series of calls with a regular spacing of 150 ± 10 ms) were manually scored. RotaRod: an automated apparatus (TSE Systems, Germany) was used to test young (2.5–5 months) and older (5.5–8 months) mice. On the first day mice were subjected to a constant speed (3 rpm) protocol for 4 sequential trials of 300 s each with inter-trial interval of 1 h 30 min. Second, for five consecutive days mice were subjected to an incremental rotation speed paradigm (3–30 rpm) in 300 s, three times a day with inter-trial interval of 1 h 30 min; the best performance of the day was selected for every single mouse to assess motor ability, coordination and learning. Hanging: mice were hanged with the forelegs to a thin metal bar and stay time was measured. Grip: a grip strength meter (Harvard Apparatus) was used to test muscle strength of the animals. Mice were allowed to grab a metal grid with the forepaws only or with all 4 paws and were gently and constantly pulled by the tail until they lost the grip. The pulling strength was measured in Newton. Three measurements were taken for each mouse and the average was used for statistical purposes. Epileptic seizures: visible tonic and tonic–clonic seizures were triggered in a number of *Pfn2*^−/−^ mice by some sudden stimulus or stressful situation. The observations in this work, for statistical reasons, come exclusively from the mice that showed the seizures upon the opening of the cage for the regular observation of the mice or the changing of the bedding. When the epileptic mouse started to become rigid, arching the back, the timer was started. Depending on the type of seizure, the mouse might remain rigid and eventually fall on one side or start convulsive movements and jumps. The timer was stopped when the mouse started to recover, as signaled by straightening and self-grooming.

### 4.3 Fluorescence immunohistochemistry

4- and 10- to12-months old mice were anesthetized and perfused with 4% formaldehyde. Brains were post-fixed O/N at 4 °C. Sagittal cerebellar slices 300 μm thick were cut with a VT1000 Vibratome (Leica Microsystems, Germany) in cold PBS. Slices were stained with antibodies following standard procedures. Anti-calbindin monoclonal antibody (Sigma, Germany, 1: 500), anti-mouse Alexa546-conjugated secondary antibody (Molecular Probes, Germany, 1:400) and the nuclear dye Hoechst (Roche, Germany, 0.5 μg/mL) were used for the staining. Imaging was performed with a Leica TSE SPE Confocal Microscope (Germany) acquiring 40 μm Z-stacks.

### 4.4 Electrophysiology

Acute hippocampal brain slices were obtained from P16-P24 mice. Animals were anesthetized with isoflurane, decapitated, the brains removed from the skull and placed into cold (4 °C) artificial cerebrospinal fluid (ACSF: 130 mM NaCl, 2.75 mM KCl, 1.43 mM MgSO_4_, 2.5 mM CaCl_2_, 1.1 mM NaH_2_PO_4_, 28.8 mM NaHCO_3_, 11 mM Glucose), pH 7.3–7.4, 315–325 mOsm/L and gassed with carbogen (95% O_2_, 5% CO_2_). The hippocampi were dissected and transversally cut in 400 μM slices with a vibratome (Leica VT1200S) in chilled and carbogenated ACSF. Slices were then equilibrated in slice-chambers containing ACSF continuously gassed with carbogen for 30 min at 32 °C and subsequently stored at room temperature in ACSF. All recordings were done at room temperature (21–23 °C) in a recording chamber continuously perfused by carbogenated ACSF. If necessary, drugs were added to the ACSF. Recordings were obtained using 2–4 MΩ glass electrodes (Science Products GB150TF-8P, Hoffenheim, Germany) filled with intracellular solution and a Multiclamp 700B amplifier (Axon Instruments), and data were digitized using a Digidata 1322A (Axon Instruments). mIPSCs: miniature inhibitory post-synaptic currents (mIPSCs) were recorded from CA1 pyramidal neurons at −70 mV holding potential for 10 min and analyzing 2 min (ca. 120 events) in the middle region of the recording, using a high chloride intracellular solution containing (in mM): 90 CsCl, 20 Cs-gluconate, 8 NaCl, 2 MgCl_2_, 10 HEPES, 1 EGTA, and 2 QX-314, pH 7.2 (290 mOsM/L). mIPSCs were recorded in ASCF supplemented with NBQX (10 μM) to block AMPA receptors and TTX (0.2 μM) to block action potentials. Evoked IPSCs: IPSCs were evoked with a stimulation electrode placed ca. 100 μm away from the soma of the recorded pyramidal cell. The position of the stimulation electrode was adjusted such that the smallest stimulus (5 μA) elicited a current of ca. 500 pA. mEPSCs: mEPSCs were recorded from CA1 pyramidal neurons in acute hippocampal brain slices at a holding potential of −70 mV, for at least 10 min to make sure that 120 events could be analyzed using the following intracellular solution (in mM): 150 Cs-gluconate, 8 NaCl, 2 MgATP, 10 HEPES, 0.2 EGTA and 5 QX-314 ([2-[(2,6-dimethylphenyl)amino]2-oxoethyl]-triethylazanium bromide), pH 7.2 (290 mOsM/L). mEPSCs were recorded in ASCF supplemented with picrotoxin (PTX, 100 μM) to block GABAA receptors, tetrodotoxin (TTX, 0.2 μM) to block action potentials and trichlormethiazide (TCM, 250 μM) to increase mEPSC frequency. fEPSCs: fEPSP were recorded from acute hippocampal slices, disconnecting the Schaffer collaterals cutting with a scalpel in the CA3 area to prevent recurrent spontaneous excitation. Schaffer collaterals after the cut were stimulated and synaptic responses were recorded in the stratum radiatum of the CA1 region, using glass microelectrodes filled with ACSF. fEPSCs were recorded in ASCF supplemented with picrotoxin (PTX, 100 μM) to block GABAA receptors. Before the experiment was started, recordings were registered for 20 min until fEPSP reached a stable baseline. For PPF experiments Schaffer collaterals were stimulated with a stimulus interval of 40 ms. For the input–output relation, the input was the amplitude of the fiber volley representing the strength of the action potential arriving from the Schaffer collaterals; the output was defined as the slope of the resulting fEPSP. Fiber volley amplitudes of 0.05, 0.1, 0.15, 0.2 and 0.25 mV were evoked and the resulting fEPSC plotted. All data were analyzed using Clampfit 10.2 (Molecular Devices), a custom written Matlab routine (MathWorks), and GraphPad Prism 5 (GraphPad Software). Values are given as means ± SEM.

Parasagittal cerebellar slices (250 μm) were prepared from 1-month old mice and immersed in ice-cold gassed (95% O_2_, 5% CO_2_) sucrose-based artificial cerebrospinal fluid (ACSF) containing: 87 mM NaCl, 75 mM sucrose, 2 mM KCl, 7 mM MgCl_2_, 0.5 mM CaCl_2_, 25 mM NaHCO_3_, 1.2 mM NaH_2_PO_4_ and 10 mM glucose, pH 7.4, 300–305 mOsm. Slices were then allowed to recover for at least 1 h at RT in standard ACSF (125 mM NaCl, 2 mM KCl, 1.2 mM MgCl_2_, 2 mM CaCl_2_, 25 mM NaHCO_3_, 1.2 mM NaH_2_PO_4_ and 10 mM glucose) before measurement. In high [Ca^2+^] experiments, [Ca^2+^] concentration was raised to 4 mM and Mg^2+^ concentration was adjusted to 1 mM. Patch-clamp recording: whole-cell patch-clamp recordings on PCs were performed with a Multiclamp 700B amplifier (Molecular Devices, USA) using glass electrodes (3–4 MΩ) pulled with a vertical puller (PC-10, Narishige, Japan). Intracellular solution (140 mM Cs-Methanesulfonate, 10 mM Hepes, 0.5 mM EGTA, 2 mM MgATP, 0.3 mM Na_3_GTP, 2 mM MgCl_2_) was adjusted to 295–300 mOsm, pH 7.2. Signals were acquired with the DigiData-1440A (AutoMate Scientific, USA) amplifier using pCLAMP-v10 software and analyzed off-line with Clamp-fit 10 program (Axon Instruments, USA). Excitatory post-synaptic currents (EPSCs) were recorded clamping the cell at −70 mV. Spontaneous EPSCs were recorded during an initial 10 min baseline period followed by application of bicuculline (10 μM, Sigma Aldrich, USA) for 15 min. A concentric bipolar stimulating electrode (SNE-100 × 50 mm long Hugo Sachs Elektronik - Harvard Apparatus GmbH, Germany) was placed in the molecular layer of the cerebellar cortex for parallel fiber stimulation. Series resistance (Rs) was not compensated during voltage-clamp experiments to avoid increased electrical noise in the trace. Rs was constantly monitored over time and recordings in which it changed more than 20% were discarded. For Paired-Pulse experiments, pairs of stimuli were applied every 20 s. Stimulus was delivered through an A320R Isostim Stimulator/Isolator (WPI, USA). PPR was calculated as the ratio between the amplitude evoked by the second stimulus (A2) and the amplitude of the first (A1; A2/A1).

### 4.5 Statistics

In behavioral tests one-way ANOVA or two-way ANOVA with repeated-measures in one factor were applied to assess statistical differences, followed by Tukey’s multiple comparisons *post hoc* test. Unpaired two-tailed t-Student’s test was used on two sets of data with normal distributions, while the non-parametric Mann–Whitney test for independent measures was used if distributions were not normal. Input–output experiments were subjected to regression analysis. Events and amplitudes frequency distributions were compared with the non-parametric Kolmogorov–Smirnov two-sample test by stochastically sampling a select number of values from each cell of the same genotype to produce a single distribution per genotype and reiterating the process 20 times, selecting at the end the highest *p* value resulting from all the comparisons. Kaplan-Mayer survival curves were analyzed applying the non-parametric Peto-Prentice generalized Wilcoxon test.

## Data Availability

The original contributions presented in the study are included in the article/Supplementary material, further inquiries can be directed to the corresponding author.
