# Supplementary material for: Autism spectrum disorder related phenotypes in a mouse model lacking the neuronal actin binding protein profilin 2
Source: Front Cell Neurosci. 2025 Feb 26;19:1540989. doi: 10.3389/fncel.2025.1540989 (PMC11897305; doi:10.3389/fncel.2025.1540989)
Supplement: Supplementary file 1 [file Table_1.docx]

Supplementary Material

# Supplementary Material and Methods

## Behavior. Nesting. Pregnant females were separated from the male and provided with fresh cotton for nesting in the last days of pregnancy. Nest quality was assessed two to three days after delivery of the litter gently opening the cage and taking a photo. Hindlimb clasping. Adult male mice were picked up at the base of the tail and suspended for 10 s evaluating the position of the limbs (Guyenet et al., 2010). Only a few mice were documented by taking a picture, as examples. Foot-printing. A4 150 g/m^2^ white drawing paper sheets were cut in 7 cm wide strips. Male mice were quickly painted under the hind paws with green ink and under the fore paws with black ink and placed on the paper strip, where they ran ahead leaving the colored footprints.

#
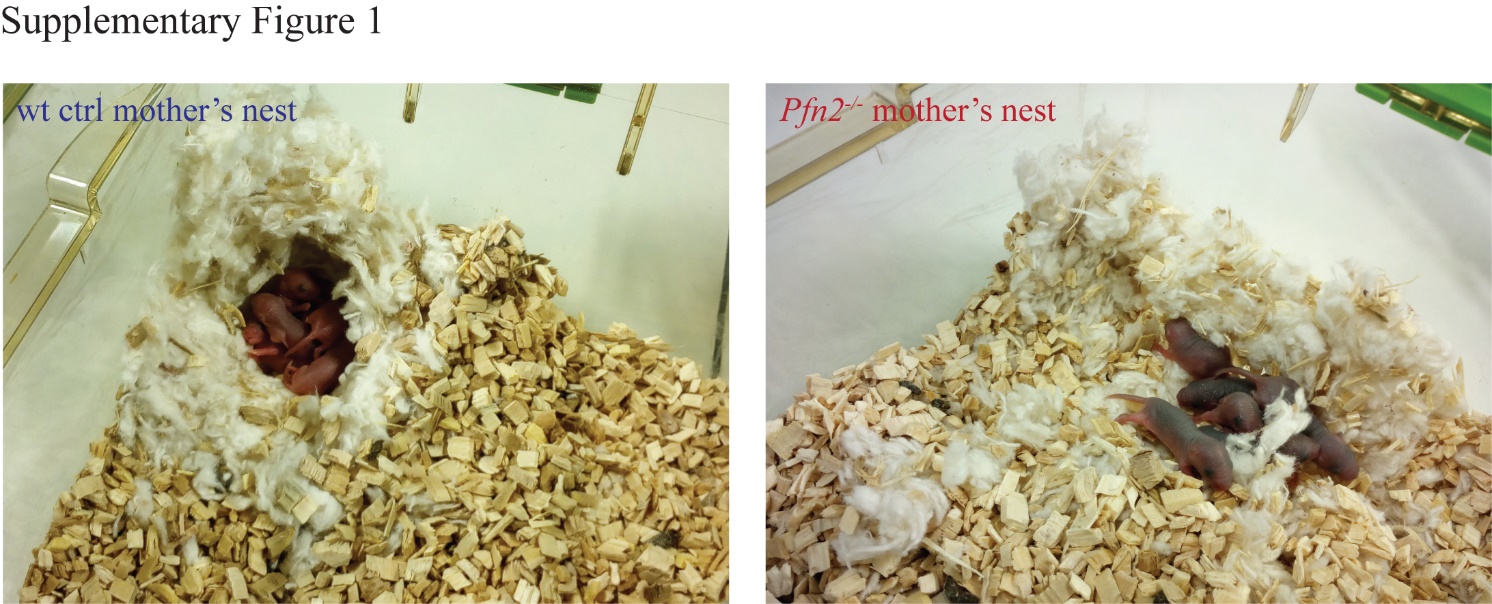
Supplementary Figures

**Supplementary Figure 1.** Nesting behavior is impaired in *Pfn2*^-/-^ females. Example of nests from wt control and *Pfn2*^-/-^ mothers, as indicated. The *Pfn2* mutant mothers did not build proper nests, leaving the nesting material and pups sparsely distributed in the cage.


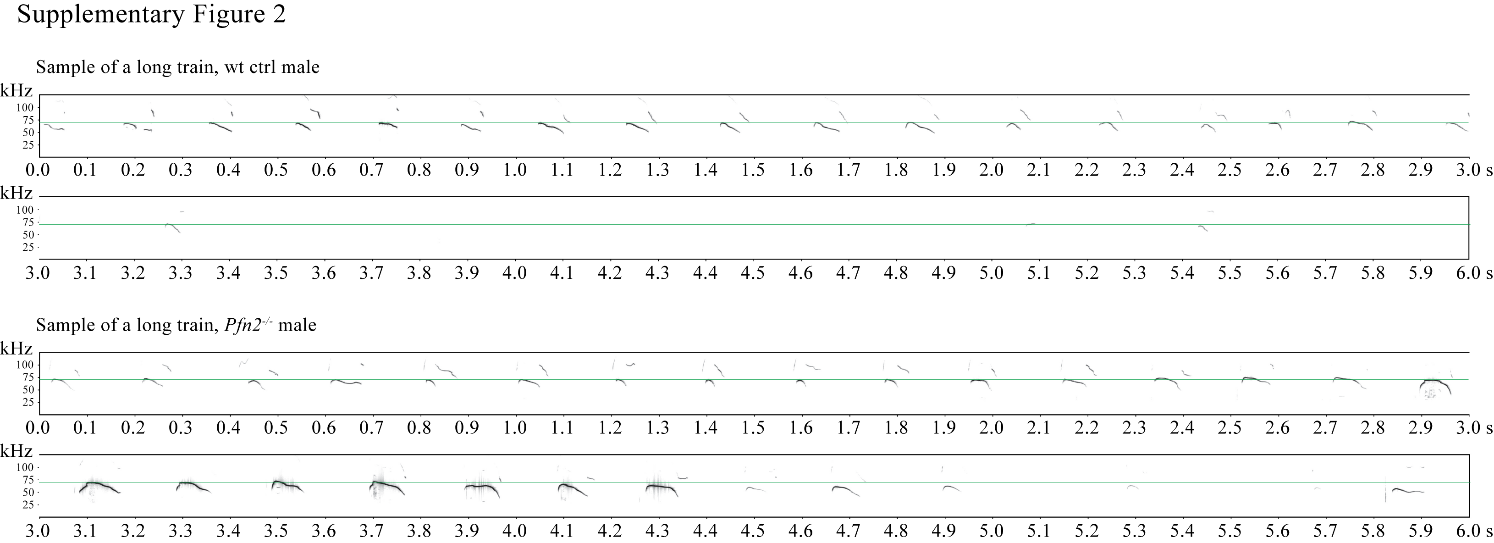
**Supplementary Figure 2.** Longer trains of flat calls in *Pfn2*^-/-^ pups separated from the mother. Samples of USV recordings (6 s) showing long trains of flat calls from male wt control and *Pfn2*^-/-^ pups separated from the mother and littermates, as indicated. USV trains from *Pfn2*^-/-^ pups are much longer than those from wt controls, with the flat calls being very intense (darker color in the gray scale). Similar patterns were observed in female pups.


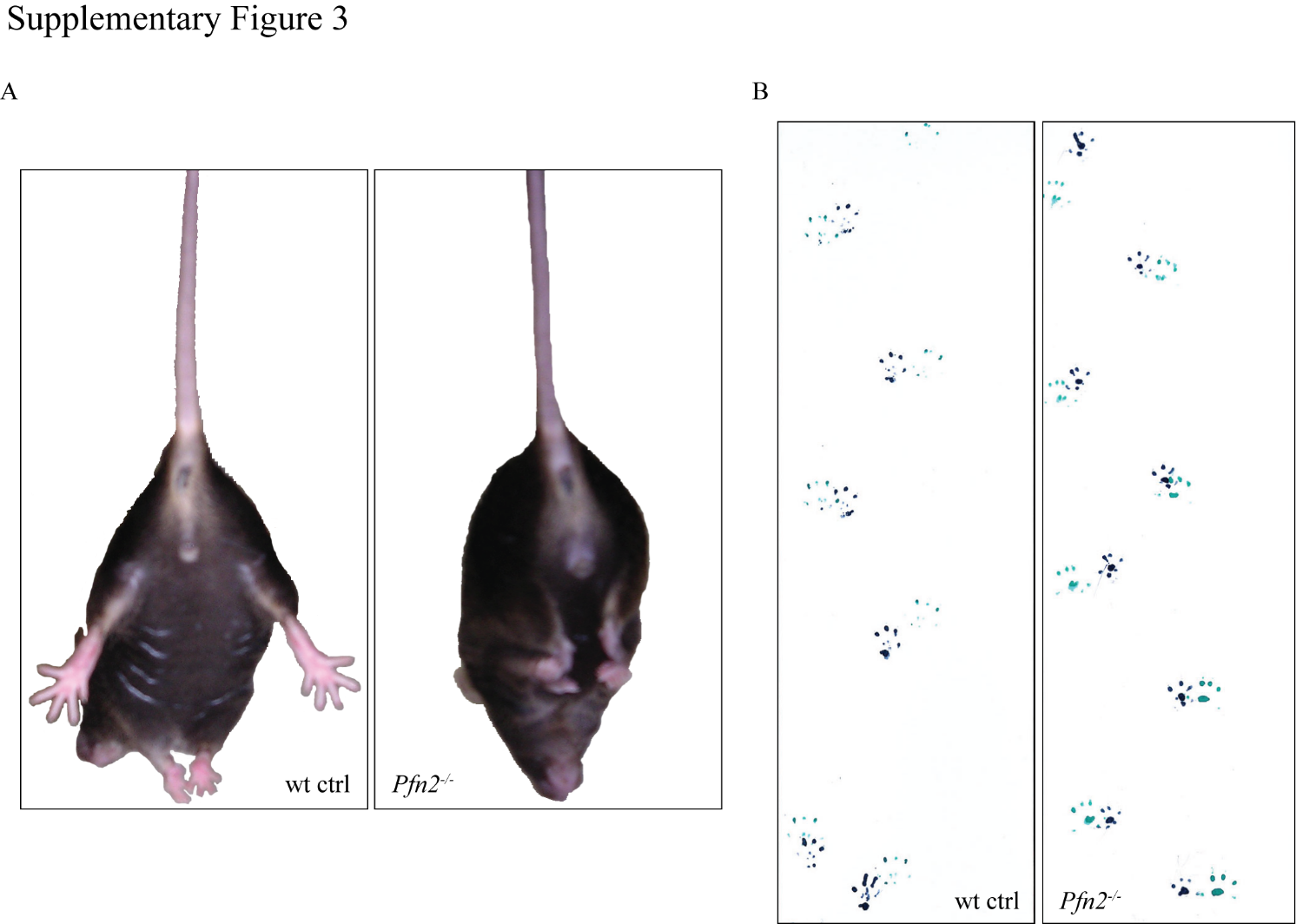


**Supplementary Figure 3.** *Pfn2*^-/-^ mice show hindlimbs clasping but do not have ataxia. (**A**) *Pfn2*^-/-^ mice show from very early age ( ̴ P10) the characteristic coordination deficit of clasping their hindlimbs when gently lifted by the tail instead of splaying them outwardly. They often also assume the bat-like position, as pictured here. (**B**) Sample tracks from foot-printing experiments on wt control and *Pfn2*^-/-^ mice at 4 months of age. There were no significant differences in the gait, except for a slightly shorter pace of the *Pfn2* mutants. Black ink: fore paws; Green ink: hind paws.


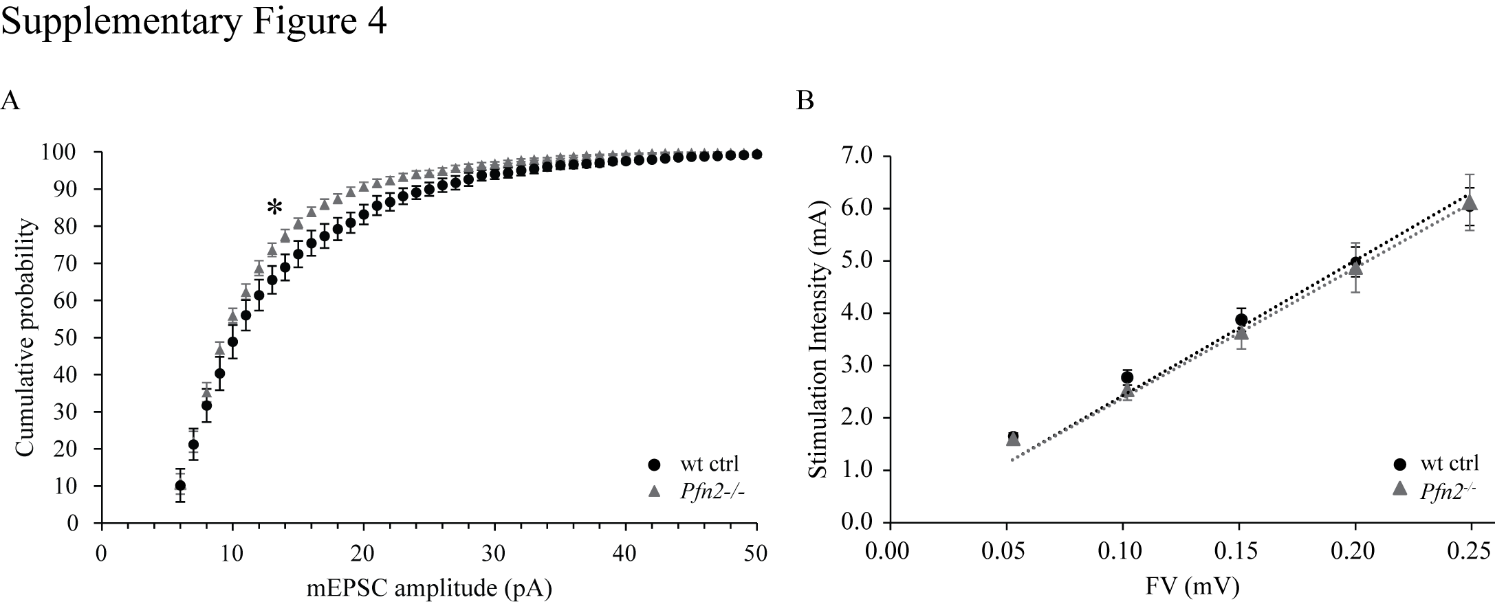


**Supplementary Figure 4.** Decreased amplitudes of miniature excitatory currents and unaltered fiber volley requirement in *Pfn2^-/-^* mice. (**A**) Percentage cumulative frequency plot of mEPSC amplitudes showed a significant decrease of average miniature current amplitudes (leftward shift) in *Pfn2^-/-^* mice compared to wt controls (Kolmogorov-Smirnov P=0.025). N=7/4 wt ctrl and n=8/3 *Pfn2^-/-^* cells/mice. (**B**) Stimulation intensity to fiber volley relationship in the excitatory I-O assay showed that axonal properties are not altered in *Pfn2^-/-^* mice. N=19/7 ctrl and n=14/7 *Pfn2^-/-^* slices/mice.

# References

Guyenet, S. J., Furrer, S. A., Damian, V. M., Baughan, T. D., La Spada, A. R., and Garden, G. A. (2010). A Simple Composite Phenotype Scoring System for Evaluating Mouse Models of Cerebellar Ataxia. *J Vis Exp*, 1787. doi: 10.3791/1787
